# Supplementary material for: Cerebellar modulation of memory encoding in the periaqueductal grey and fear behaviour
Source: eLife. 2022 Mar 15;11:e76278. doi: 10.7554/eLife.76278 (PMC8923669; doi:10.7554/eLife.76278)
Supplement: Figure 1—figure supplement 1—source data 1. [file elife-76278-fig1-figsupp1-data1.docx]

|  |  |  |  |  |
| --- | --- | --- | --- | --- |

**Figure 1 – figure supplement 1**.

**Tetrode recordings.**

| **C. Firing rates for controls**  Individual data points showing the firing rate for each individual unit (Hz) | | |  | **D. Firing rates for muscimol**  Individual data points showing the firing rate for each individual unit (Hz) | | |
| --- | --- | --- | --- | --- | --- | --- |
| **Control** | | |  | **Muscimol** | | |
| **Habituation** | **Conditioning** | **Retrieval** |  | **Habituation** | **Conditioning** | **Retrieval** |
| 2.30 | 13.60 | 15.10 |  | 27.58 | 4.11 | 8.33 |
| 3.50 | 20.20 | 5.11 |  | 8.38 | 13.47 | 3.28 |
| 2.60 | 3.30 | 3.90 |  | 9.56 | 2.54 | 3.78 |
| 2.70 | 2.60 | 4.90 |  | 3.59 | 45.32 | 49.99 |
| 1.70 | 9.87 | 4.80 |  | 17.52 | 1.62 | 1.70 |
| 17.73 | 6.38 | 7.90 |  | 3.98 | 1.41 | 5.62 |
| 6.76 | 44.95 | 56.74 |  | 17.11 | 0.50 | 2.69 |
| 48.36 | 11.12 | 16.56 |  | 13.34 | 0.98 | 4.95 |
| 11.38 | 11.98 | 11.84 |  | 26.92 | 4.08 | 4.18 |
| 12.99 | 7.38 | 39.40 |  | 34.95 | 3.26 | 4.44 |
| 19.75 | 25.39 | 1.27 |  |  | 6.49 | 3.11 |
| 17.89 | 1.67 | 3.20 |  |  | 7.89 | 10.80 |
| 3.15 | 2.34 | 7.19 |  |  | 4.47 | 6.34 |
| 11.09 | 4.56 | 10.34 |  |  | 5.08 | 20.69 |
| 4.64 | 4.27 | 1.46 |  |  | 1.95 | 25.70 |
| 5.49 | 2.71 | 2.61 |  |  | 2.86 | 7.20 |
| 10.62 | 6.75 | 4.77 |  |  | 13.14 | 17.45 |
| 3.22 | 2.21 | 2.56 |  |  |  | 5.40 |
| 19.32 | 2.17 | 9.32 |  |  |  | 0.78 |
| 0.30 | 1.35 | 9.35 |  |  |  | 0.98 |
| 1.50 | 0.47 | 6.45 |  |  |  | 1.05 |
| 1.36 | 0.39 | 5.75 |  |  |  | 1.41 |
| 1.61 | 2.70 | 4.13 |  |  |  | 8.10 |
| 6.40 | 1.59 | 15.01 |  |  |  | 1.53 |
| 2.29 | 2.81 | 2.05 |  |  |  | 5.87 |
| 15.84 | 1.33 | 0.85 |  |  |  |  |
| 4.06 | 4.99 | 2.28 |  |  |  |  |
| 5.56 | 8.27 | 1.09 |  |  |  |  |
| 4.31 | 0.76 | 0.69 |  |  |  |  |
| 4.48 | 11.03 | 1.76 |  |  |  |  |
|  | 11.45 | 20.26 |  |  |  |  |
|  | 6.06 | 1.97 |  |  |  |  |
|  | 4.25 | 3.48 |  |  |  |  |
|  | 8.55 | 3.25 |  |  |  |  |
|  | 12.89 | 3.78 |  |  |  |  |
|  | 0.58 | 3.08 |  |  |  |  |
|  | 4.95 | 9.20 |  |  |  |  |
|  | 9.80 | 16.52 |  |  |  |  |
|  | 4.58 | 10.57 |  |  |  |  |
|  | 4.11 | 4.12 |  |  |  |  |
|  | 6.59 | 11.74 |  |  |  |  |
|  | 5.61 | 1.73 |  |  |  |  |
|  | 2.83 | 0.50 |  |  |  |  |
|  | 6.86 | 1.44 |  |  |  |  |
|  | 11.34 | 3.27 |  |  |  |  |
|  | 3.26 | 3.27 |  |  |  |  |
|  | 4.04 | 1.57 |  |  |  |  |
|  | 1.12 | 7.25 |  |  |  |  |
|  | 4.16 | 5.35 |  |  |  |  |
|  | 4.79 | 7.06 |  |  |  |  |
|  |  | 7.44 |  |  |  |  |
|  |  | 3.34 |  |  |  |  |
|  |  | 2.95 |  |  |  |  |
|  |  | 3.69 |  |  |  |  |
|  |  | 3.61 |  |  |  |  |
