# Supplementary material for: Cerebellar modulation of memory encoding in the periaqueductal grey and fear behaviour
Source: eLife. 2022 Mar 15;11:e76278. doi: 10.7554/eLife.76278 (PMC8923669; doi:10.7554/eLife.76278)
Supplement: Figure 1—figure supplement 3—source data 1. [file elife-76278-fig1-figsupp3-data1.docx]

**Figure 1 – figure supplement 3.**

**vlPAG offset responses during auditory cued fear trace conditioning.**

| **E. Latency to peak or trough**  Individual data points showing the latency to peak or trough per individual unit (ms) | |
| --- | --- |
| **Delay** | **Trace Type I** |
| 0 | 40 |
| 40 | 80 |
| 40 | 120 |
| 280 | 40 |
| 80 | 160 |
| 0 | 0 |
| 0 |  |
| 0 | **Trace Type IV** |
| 160 | 160 |
| 0 | 160 |
| 0 | 40 |
| 80 | 40 |
| 0 | 80 |
| 80 | 120 |
