# Supplementary material for: Cerebellar modulation of memory encoding in the periaqueductal grey and fear behaviour
Source: eLife. 2022 Mar 15;11:e76278. doi: 10.7554/eLife.76278 (PMC8923669; doi:10.7554/eLife.76278)
Supplement: Figure 2—source data 1. [file elife-76278-fig2-data1.docx]

**Figure 2.**

**Effect of MCN inactivation during consolidation on vPAG type 1 onset and offset responses during extinction.**

| **D. Response area of type 1 onset units**  Individual data points of single unit type 1 onset response areas (a.u) during EE and LE in control versus muscimol treated animals. | | | |  |
| --- | --- | --- | --- | --- |
|  |  |  |  |  |
| **Control EE** | **Muscimol EE** | **Control LE** | **Muscimol LE** |  |
| 5.07 | 79.82 | -2.09 | 26.49 |  |
| 197.92 | 67.76 | 173.95 | 45.92 |  |
| 48.91 | 11.25 | 36.91 | -0.49 |  |
| 42.23 | 18.75 | 23.67 | -2.31 |  |
| 24.19 | 21.89 | 3.46 | -1.05 |  |
| 46.53 | 34.26 | 3.84 | 21.91 |  |
| 14.41 | 51.73 | -4.44 | -5.20 |  |
| 21.23 | 51.59 | 5.87 | -13.09 |  |
| 12.10 | 4.57 | -1.64 | -5.27 |  |
| 25.56 | 15.31 | 10.72 | 3.06 |  |
| 12.82 |  | -1.95 |  |  |
| 61.14 |  | 49.17 |  |  |
| 17.09 |  | 13.95 |  |  |
| 15.94 |  | 8.26 |  |  |
| 51.15 |  | -9.72 |  |  |
| 7.31 |  | -2.65 |  |  |
| 38.64 |  | 34.32 |  |  |
| 30.61 |  | 22.71 |  |  |

| **E. The z-score peak firing rate of type 1 onset**  Individual data points showing the z-scored average type 1 onset peak response during EE and LE in control versus muscimol treated animals. | | | |  |
| --- | --- | --- | --- | --- |
|  |  |  |  |  |
| **Control EE** | **Muscimol EE** | **Control LE** | **Muscimol LE** |  |
| 2.12 | 5.58 | 3.08 | 5.39 |  |
| 33.42 | 9.17 | 33.72 | 6.53 |  |
| 8.95 | -1.20 | 5.21 | 1.71 |  |
| 0.82 | 4.55 | 0.74 | 0.50 |  |
| 0.40 | 2.85 | 1.02 | 1.83 |  |
| 4.61 | 5.02 | 2.94 | 2.95 |  |
| -0.96 | 3.61 | 0.82 | -0.12 |  |
| 1.93 | -0.31 | 1.3 | 2.70 |  |
| 0.08 | 4.52 | 0.01 | 0.32 |  |
| 1.99 | 6.19 | 1.78 | 3.94 |  |
| -0.98 |  | -0.89 |  |  |
| 8.53 |  | 6.4 |  |  |
| 4.87 |  | 1.47 |  |  |
| 0.95 |  | 0.28 |  |  |
| 0.56 |  | 2.23 |  |  |
| 6.17 |  | 2.25 |  |  |
| 1.77 |  | 2.62 |  |  |
| 1.56 |  | 1.85 |  |  |

| **H. Response area of type 1 offset units**  Individual data points of single unit type 1 offset response areas (a.u) during EE and LE in control versus muscimol animals. | | | |  |
| --- | --- | --- | --- | --- |
|  |  |  |  |  |
| **Control EE** | **Muscimol EE** | **Control LE** | **Muscimol LE** |  |
| 57.76 | 59.11 | 28.35 | 20.79 |  |
| 86.56 | 70.31 | 20.26 | 14.72 |  |
| 2.95 | 35.25 | -1.04 | 28.93 |  |
| 6.37 | 21.15 | 4.25 | 3.17 |  |
| -1.24 | 18.40 | -7.55 | 2.25 |  |
| 28.23 | 26.47 | -4.56 | -3.11 |  |
| -2.38 | 27.70 | -9.17 | 7.93 |  |
| 16.55 | 73.74 | -15.05 | 19.17 |  |
| 2.09 | 70.69 | -23.06 | -13.09 |  |
| 18.04 | 28.01 | -18.12 | 14.83 |  |
| -4.97 |  | -18.43 |  |  |
| 63.80 |  | -19.23 |  |  |
| 14.36 |  | 11.28 |  |  |
| 21.89 |  | -5.51 |  |  |
| 13.37 |  | 4.56 |  |  |
| 12.82 |  | -10.03 |  |  |
| -5.64 |  | -12.76 |  |  |
| -0.15 |  | -11.13 |  |  |
| 9.66 |  | 5.99 |  |  |

| **I. The z-score peak firing rate of type 1 offset**  Individual data points showing the z-scored average type 1 offset peak response during EE and LE in control versus muscimol animals. | | | |  |
| --- | --- | --- | --- | --- |
|  |  |  |  |  |
| **Control EE** | **Muscimol EE** | **Control LE** | **Muscimol LE** |  |
| 3.05 | 2.40 | 3.24 | 4.13 |  |
| 42.70 | 10.44 | 14.76 | 9.02 |  |
| 4.41 | 3.57 | 1.20 | 2.43 |  |
| -0.33 | 1.18 | -0.09 | 1.71 |  |
| 1.64 | 1.81 | -0.55 | 2.11 |  |
| 3.84 | 2.15 | 1.74 | 1.83 |  |
| 0.12 | 2.83 | 1.77 | 2.38 |  |
| 8.18 | 3.61 | 1.30 | 1.20 |  |
| 1.83 | 0.47 | 0.01 | -0.61 |  |
| 0.35 | 2.84 | -1.63 | 6.88 |  |
| -0.98 |  | -0.89 |  |  |
| 12.86 |  | 6.40 |  |  |
| 7.22 |  | 1.77 |  |  |
| 3.13 |  | -0.73 |  |  |
| 6.82 |  | 2.75 |  |  |
| 0.62 |  | 1.84 |  |  |
| 1.37 |  | 1.55 |  |  |
| 1.69 |  | -0.40 |  |  |
| 0.64 |  | -0.66 |  |  |
