# Supplementary material for: Cerebellar modulation of memory encoding in the periaqueductal grey and fear behaviour
Source: eLife. 2022 Mar 15;11:e76278. doi: 10.7554/eLife.76278 (PMC8923669; doi:10.7554/eLife.76278)
Supplement: Figure 2—figure supplement 1—source data 1. [file elife-76278-fig2-figsupp1-data1.docx]

**Figure 2 – figure supplement 1.**

**Comparison of single unit results for tetrodes only versus saline control animals.**

| **C. Response area at CS+ onset**  Individual data points showing the mean response area per unit (a.u.) | | | |  | **F. Response area at CS+ offset**  Individual data points showing the mean response area per unit (a.u.) | | | |
| --- | --- | --- | --- | --- | --- | --- | --- | --- |
| **Tetrode EE** | **Saline EE** | **Tetrode LE** | **Saline LE** |  | **Tetrode EE** | **Saline EE** | **Tetrode LE** | **Saline LE** |
| 5.07 | 51.15 | -2.09 | -9.72 |  | 57.76 | -5.64 | 28.35 | -12.76 |
| 197.92 | 7.31 | 173.95 | -2.65 |  | 86.56 | -0.15 | 20.26 | -11.13 |
| 48.91 | 38.64 | 36.91 | 34.32 |  | 2.95 | 9.66 | -1.04 | 5.99 |
| 42.23 | 30.61 | 23.67 | 22.71 |  | 6.37 |  | 4.25 |  |
| 24.19 |  | 3.46 |  |  | -1.24 |  | -7.55 |  |
| 46.53 |  | 3.84 |  |  | 28.23 |  | -4.56 |  |
| 14.41 |  | -4.44 |  |  | -2.38 |  | -9.17 |  |
| 21.23 |  | 5.87 |  |  | 16.55 |  | -15.05 |  |
| 12.10 |  | -1.64 |  |  | 2.09 |  | -23.06 |  |
| 25.56 |  | 10.72 |  |  | 18.04 |  | -18.12 |  |
| 12.82 |  | -1.95 |  |  | -4.97 |  | -18.43 |  |
| 61.14 |  | 49.17 |  |  | 63.80 |  | -19.23 |  |
| 17.09 |  | 13.95 |  |  | 14.36 |  | 11.28 |  |
| 15.94 |  | 8.26 |  |  | 21.89 |  | -5.51 |  |
|  |  |  |  |  | 13.37 |  | 4.56 |  |
|  |  |  |  |  | 12.82 |  | -10.03 |  |
