# Supplementary material for: Cerebellar modulation of memory encoding in the periaqueductal grey and fear behaviour
Source: eLife. 2022 Mar 15;11:e76278. doi: 10.7554/eLife.76278 (PMC8923669; doi:10.7554/eLife.76278)
Supplement: Figure 3—source data 1. [file elife-76278-fig3-data1.docx]

**Figure 3.**

**Auditory event related field potentials (ERPs) recorded simultaneously in the MCN and vlPAG during extinction.**

| **C and D. ERPs peak to peak amplitude.**  Individual data points showing mean peak to peak amplitude (µV) of ERPs recorded at CS+ onset and offset in EE versus LE. | | | | | | | | | | |
| --- | --- | --- | --- | --- | --- | --- | --- | --- | --- | --- |
| **MCN CS+ onset** | |  | **vlPAG CS+ onset** | |  | **MCN CS+ offset** | |  | **vlPAG CS+ offset** | |
| **EE** | **LE** |  | **EE** | **LE** |  | **EE** | **LE** |  | **EE** | **LE** |
| 240.70 | 274.23 |  | 282.64 | 288.30 |  | 268.92 | 249.07 |  | 219.47 | 203.02 |
| 208.55 | 163.84 |  | 310.51 | 173.21 |  | 362.09 | 270.00 |  | 594.15 | 327.93 |
| 489.07 | 251.28 |  | 636.20 | 396.86 |  | 299.12 | 181.92 |  | 336.77 | 170.09 |
| 367.97 | 158.10 |  | 419.10 | 318.87 |  | 573.09 | 409.15 |  | 390.97 | 272.57 |
| 1010.67 | 740.21 |  | 958.52 | 700.98 |  | 1375.44 | 1085.43 |  | 1085.67 | 806.04 |
| 212.55 | 267.98 |  | 399.61 | 630.04 |  | 345.01 | 387.65 |  | 494.60 | 247.05 |

| **E. Repeated measures correlation for ERPs at CS+ onset**  Individual data points showing peak to peak amplitude (µV) | | | |  | **F. Repeated measures correlation for ERPs at CS+ offset**  Individual data points showing peak to peak amplitude (µV) | | | |
| --- | --- | --- | --- | --- | --- | --- | --- | --- |
| **Animal ID** | **Block n** | **MCN** | **vPAG** |  | **Animal ID** | **Block n** | **MCN** | **vPAG** |
| 1 | 1 | 225.13 | 367.1 |  | 1 | 1 | 368 | 361.6 |
| 1 | 2 | 222.39 | 296.4 |  | 1 | 2 | 202.22 | 142.77 |
| 1 | 3 | 246.42 | 347.3 |  | 1 | 3 | 449.7 | 338.2 |
| 1 | 4 | 334.37 | 282.15 |  | 1 | 4 | 368.2 | 321.6 |
| 1 | 5 | 338.8 | 355.6 |  | 1 | 5 | 202.22 | 143.9 |
| 2 | 1 | 181.13 | 316.312 |  | 2 | 1 | 315.7 | 563.3 |
| 2 | 2 | 315.7 | 324.7 |  | 2 | 2 | 435.3 | 793.1 |
| 2 | 3 | 325.9 | 175.71 |  | 2 | 3 | 327.8 | 641.2 |
| 2 | 4 | 187.89 | 146.6793 |  | 2 | 4 | 210.35 | 367.83 |
| 2 | 5 | 258.58 | 362.9 |  | 2 | 5 | 335.1 | 426.7 |
| 3 | 1 | 495.25 | 675.1 |  | 3 | 1 | 313.24 | 251.718 |
| 3 | 2 | 461.46 | 600.5 |  | 3 | 2 | 319.54 | 332.82 |
| 3 | 3 | 471.5 | 544.3 |  | 3 | 3 | 348.8 | 268.86 |
| 3 | 4 | 231.62 | 294.89 |  | 3 | 4 | 93.17 | 266.7784 |
| 3 | 5 | 333.3 | 460.7 |  | 3 | 5 | 259.3 | 184.37 |
| 4 | 1 | 327.6 | 376.45 |  | 4 | 1 | 552.6 | 588.6 |
| 4 | 2 | 428.5 | 502.5 |  | 4 | 2 | 631.2 | 639.4 |
| 4 | 3 | 220.62 | 343.3 |  | 4 | 3 | 512.6 | 592.7 |
| 4 | 4 | 207.48 | 285.6 |  | 4 | 4 | 345.5 | 525.9 |
| 5 | 1 | 719 | 792 |  | 5 | 1 | 1182.2 | 1032.3 |
| 5 | 2 | 1330 | 1142.9 |  | 5 | 2 | 1578.5 | 1199 |
| 5 | 3 | 1327.3 | 1225.4 |  | 5 | 3 | 1444.3 | 1184.7 |
| 5 | 4 | 784.3 | 706.6 |  | 5 | 4 | 1003.4 | 920.7 |
| 5 | 5 | 759.7 | 789.3 |  | 5 | 5 | 1224 | 838.1 |
| 6 | 1 | 285.07 | 495.22 |  | 6 | 1 | 243.81 | 469.9 |
| 6 | 2 | 161.384 | 312.1 |  | 6 | 2 | 303.3 | 567.7 |
| 6 | 3 | 297.6 | 520.3 |  | 6 | 3 | 276.69 | 420.4 |
| 6 | 4 | 161.7 | 533.3 |  | 6 | 4 | 457 | 350.1 |
| 6 | 5 | 405.1 | 716.6 |  | 6 | 5 | 304.32 | 189.38 |
