# Supplementary material for: Cerebellar modulation of memory encoding in the periaqueductal grey and fear behaviour
Source: eLife. 2022 Mar 15;11:e76278. doi: 10.7554/eLife.76278 (PMC8923669; doi:10.7554/eLife.76278)
Supplement: Figure 4—source data 1. [file elife-76278-fig4-data1.docx]

**Figure 4.**

**Effect of MCN inactivation during consolidation on the timing of vlPAG type 1 onset and offset responses during extinction.**

| **A. The number of significant peaks after tone onset.**  Individual data points showing number of significant peaks per single unit (n) during the initial 500 ms following tone onset. | | | |  | **B. The proportion of maximum peak responses over time at tone onset**  Proportion of maximum peaks from each animal per time bin (%) | | |
| --- | --- | --- | --- | --- | --- | --- | --- |
| **Control EE** | **Control LE** | **Muscimol EE** | **Muscimol LE** |  | **Time Bin** | **Control** | **Muscimol** |
| 3 | 2 | 1 | 3 |  | 1-100 | 57.14 | 57.14 |
| 1 | 3 | 1 | 2 |  | 101-200 | 28.57 | 21.43 |
| 1 | 1 | 2 | 3 |  | 201-300 | 0.00 | 7.14 |
| 1 | 1 | 2 | 1 |  | 301-400 | 9.52 | 14.29 |
| 3 | 2 | 3 | 1 |  | 401-500 | 4.76 | 0.00 |
| 2 | 1 | 1 | 1 |  |  |  |  |
| 1 | 1 | 3 | 3 |  |  |  |  |
| 1 | 2 | 3 | 0 |  |  |  |  |
| 3 | 1 | 4 | 1 |  |  |  |  |
| 2 | 1 | 2 | 1 |  |  |  |  |
| 1 | 1 | 2 | 1 |  |  |  |  |
| 2 | 0 | 2 | 2 |  |  |  |  |
| 0 | 0 | 1 | 0 |  |  |  |  |
| 1 | 1 | 2 | 1 |  |  |  |  |
| 0 | 2 |  | 2 |  |  |  |  |
| 3 | 2 |  |  |  |  |  |  |
| 2 | 1 |  |  |  |  |  |  |
| 1 | 2 |  |  |  |  |  |  |
| 2 | 1 |  |  |  |  |  |  |
| 1 | 1 |  |  |  |  |  |  |
| 1 | 1 |  |  |  |  |  |  |
| 3 | 1 |  |  |  |  |  |  |
| 2 | 2 |  |  |  |  |  |  |
| 1 | 1 |  |  |  |  |  |  |

| **C. Number of significant peaks in the initial 500 ms after offset**  Individual data points showing number of significant peaks per single unit (n) during the initial 500 ms following tone onset. | | | |  | **D. Proportion of maximum peak responses over time at offset**  Proportion of maximum peaks from each animal per time bin (%) | | |
| --- | --- | --- | --- | --- | --- | --- | --- |
| **Control EE** | **Control LE** | **Muscimol EE** | **Muscimol LE** |  | **Time Bin** | **Control** | **Muscimol** |
| 1 | 4 | 3 | 2 |  | 1-100 | 45.45 | 10 |
| 2 | 3 | 4 | 2 |  | 101-200 | 36.36 | 10 |
| 1 | 1 | 4 | 3 |  | 201-300 | 9.091 | 10 |
| 1 | 1 | 1 | 1 |  | 301-400 | 4.545 | 30 |
| 1 | 1 | 2 | 1 |  | 401-500 | 4.545 | 40 |
| 1 | 1 | 2 | 1 |  |  |  |  |
| 1 | 1 | 3 | 1 |  |  |  |  |
| 1 | 1 | 3 | 2 |  |  |  |  |
| 1 | 1 | 3 | 1 |  |  |  |  |
| 1 | 1 | 2 | 1 |  |  |  |  |
| 1 | 1 |  | 2 |  |  |  |  |
| 1 | 1 |  |  |  |  |  |  |
| 0 | 3 |  |  |  |  |  |  |
| 1 |  |  |  |  |  |  |  |
| 1 |  |  |  |  |  |  |  |
| 1 |  |  |  |  |  |  |  |
| 1 |  |  |  |  |  |  |  |
| 1 |  |  |  |  |  |  |  |
| 2 |  |  |  |  |  |  |  |
| 1 |  |  |  |  |  |  |  |
| 1 |  |  |  |  |  |  |  |
| 1 |  |  |  |  |  |  |  |
| 0 |  |  |  |  |  |  |  |
