# Supplementary material for: Cerebellar modulation of memory encoding in the periaqueductal grey and fear behaviour
Source: eLife. 2022 Mar 15;11:e76278. doi: 10.7554/eLife.76278 (PMC8923669; doi:10.7554/eLife.76278)
Supplement: Figure 5—source data 1. [file elife-76278-fig5-data1.docx]

**Figure 5.**

**Freezing activity during extinction for control and muscimol animals.**

| **A. Freezing (%) during CS+**  Individual data points showing the percentage of time animals displayed freezing behaviour (%) | | | |  | **B. Freezing (%) during ITI**  Individual data points showing the percentage of time animals displayed freezing behaviour (%) | | | |
| --- | --- | --- | --- | --- | --- | --- | --- | --- |
| **Control EE** | **Muscimol EE** | **Control LE** | **Muscimol LE** |  | **Control EE** | **Muscimol EE** | **Control LE** | **Muscimol LE** |
| 60.00 | 51.43 | 0.00 | 8.04 |  | 39.29 | 49.58 | 0.00 | 7.50 |
| 80.00 | 70.36 | 20.71 | 55.49 |  | 82.89 | 63.38 | 9.57 | 48.18 |
| 65.36 | 68.57 | 5.00 | 10.18 |  | 74.52 | 63.03 | 12.07 | 15.98 |
| 79.64 | 67.86 | 27.86 | 28.04 |  | 75.34 | 53.69 | 10.37 | 19.35 |
| 84.64 | 99.00 | 63.21 | 72.57 |  | 82.68 | 100.00 | 64.72 | 66.95 |
| 93.57 | 78.14 | 72.86 | 5.14 |  | 97.32 | 67.67 | 76.46 | 7.76 |
| 60.36 | 58.71 | 19.64 | 26.71 |  | 35.95 | 36.67 | 4.41 | 37.86 |
| 67.50 | 99.29 | 30.00 | 81.71 |  | 54.29 | 86.43 | 22.72 | 80.52 |
| 66.07 |  | 37.50 |  |  | 59.23 |  | 21.45 |  |
| 83.93 |  | 33.93 |  |  | 53.63 |  | 19.60 |  |

| **C. Duration of freezing epochs**  Individual data points showing the average duration of time animals displayed freezing behaviour (s) | | | |  | **D. Duration of moving epochs**  Individual data points showing the average duration of time animals displayed moving epochs (s) | | | |
| --- | --- | --- | --- | --- | --- | --- | --- | --- |
| **Control EE** | **Muscimol EE** | **Control LE** | **Muscimol**  **LE** |  | **Control**  **EE** | **Muscimol EE** | **Control EE** | **Muscimol LE** |
| 2.9 | 14.9 | 0 | 7.8 |  | 1.5 | 7.3 | 2.2 | 12.1 |
| 11 | 23.1 | 5.9 | 15 |  | 6.6 | 3.5 | 3.6 | 4 |
| 11.6 | 14.4 | 2.3 | 10.4 |  | 1.6 | 2 | 9.9 | 1.3 |
| 36.4 | 7.3 | 8.125 | 5 |  | 1.4 | 4.3 | 14.1 | 2.6 |
| 24 | 155.9 | 3.4 | 34.32 |  | 1.4 | 2 | 1.2 | 22.44 |
| 7.4 | 45.6 | 9.8 | 2.4 |  | 0.9 | 3.88 | 1.6 | 12.6 |
| 1.9 | 29.8 | 1.3 | 7.44 |  | 1.9 | 7 | 15.1 | 17.12 |
| 5.6 | 94.44 | 2.2 | 26.36 |  | 2.4 | 6.24 | 6.3 | 4.92 |
| 2.6 |  | 3.3 |  |  | 1.6 |  | 4.4 |  |
| 11.6 |  | 3.4 |  |  | 1.7 |  | 3.4 |  |
|  |  |  |  |  |  |  |  |  |

| **E Top. Repeated measures correlation of CS+ onset response area vs freezing (%) during CS+ per trial in the control group**  Individual data points showing the average response area (a.u.) and freezing (%) per animal over each block (5 trials) | | | |  | **E Bottom. Repeated measures correlation of CS+ onset response area vs freezing (%) during CS+ per trial in the muscimol group**  Individual data points showing the average response area (a.u.) and freezing (%) per animal over each block (5 trials) | | | |
| --- | --- | --- | --- | --- | --- | --- | --- | --- |
| **Animal ID** | **Block (n)** | **Freezing (%)** | **Response area** |  | **Animal ID** | **Block (n)** | **Freezing (%)** | **Response area** |
| 1.00 | 1.00 | 74.29 | 9.67 |  | 1.00 | 1.00 | 55.00 | 75.40 |
| 1.00 | 2.00 | 85.71 | -2.68 |  | 1.00 | 2.00 | 47.86 | 26.26 |
| 1.00 | 3.00 | 82.14 | -13.46 |  | 1.00 | 3.00 | 5.00 | 1.92 |
| 1.00 | 4.00 | 41.43 | -19.43 |  | 1.00 | 4.00 | 10.71 | 30.23 |
| 1.00 | 5.00 | 20.71 | -9.69 |  | 1.00 | 5.00 | 5.36 | 29.27 |
| 2.00 | 1.00 | 63.57 | 77.77 |  | 2.00 | 1.00 | 90.71 | 15.35 |
| 2.00 | 2.00 | 67.14 | 60.27 |  | 2.00 | 2.00 | 50.00 | 5.76 |
| 2.00 | 3.00 | 42.86 | 78.02 |  | 2.00 | 3.00 | 66.43 | 6.99 |
| 2.00 | 4.00 | 17.14 | 37.91 |  | 2.00 | 4.00 | 52.14 | -3.36 |
| 2.00 | 5.00 | 5.00 | 74.85 |  | 2.00 | 5.00 | 58.85 | 1.78 |
| 3.00 | 1.00 | 86.43 | 28.82 |  | 3.00 | 1.00 | 68.57 | 14.65 |
| 3.00 | 2.00 | 72.86 | 21.24 |  | 3.00 | 2.00 | 68.57 | 6.61 |
| 3.00 | 3.00 | 41.43 | 5.19 |  | 3.00 | 3.00 | 38.57 | -1.98 |
| 3.00 | 4.00 | 14.29 | 6.94 |  | 3.00 | 4.00 | 13.57 | 6.26 |
| 4.00 | 1.00 | 87.14 | 5.99 |  | 3.00 | 5.00 | 6.79 | -0.95 |
| 4.00 | 2.00 | 82.14 | 28.59 |  |  |  |  |  |
| 4.00 | 3.00 | 85.00 | 8.96 |  |  |  |  |  |
| 4.00 | 4.00 | 63.57 | 11.18 |  |  |  |  |  |
| 4.00 | 5.00 | 63.21 | 3.65 |  |  |  |  |  |
| 5.00 | 1.00 | 97.14 | 1.82 |  |  |  |  |  |
| 5.00 | 2.00 | 90.00 | 18.44 |  |  |  |  |  |
| 5.00 | 3.00 | 70.00 | 3.33 |  |  |  |  |  |
| 5.00 | 4.00 | 70.71 | 21.53 |  |  |  |  |  |
| 5.00 | 5.00 | 72.86 | 2.90 |  |  |  |  |  |
| 6.00 | 1.00 | 72.14 | 24.53 |  |  |  |  |  |
| 6.00 | 2.00 | 60.00 | 21.42 |  |  |  |  |  |
| 6.00 | 3.00 | 72.14 | -5.53 |  |  |  |  |  |
| 6.00 | 4.00 | 24.29 | -1.27 |  |  |  |  |  |
| 6.00 | 5.00 | 50.71 | -5.82 |  |  |  |  |  |
| 7.00 | 1.00 | 88.57 | 27.74 |  |  |  |  |  |
| 7.00 | 2.00 | 79.29 | 22.31 |  |  |  |  |  |
| 7.00 | 3.00 | 65.00 | 18.19 |  |  |  |  |  |
| 7.00 | 4.00 | 53.57 | 30.60 |  |  |  |  |  |
| 7.00 | 5.00 | 14.29 | 16.76 |  |  |  |  |  |
|  |  |  |  |  |  |  |  |  |

| **F Top. Repeated measures correlation of CS+ offset response area vs freezing (%) during ITI per trial in the control group**  Individual data points showing the average response area (a.u.) and freezing (%) per animal over each block (5 trials) | | | |  | **E Bottom. Repeated measures correlation of CS+ offset response area vs freezing (%) during ITI per trial in the muscimol group**  Individual data points showing the average response area (a.u.) and freezing (%) per animal over each block (5 trials) | | | |
| --- | --- | --- | --- | --- | --- | --- | --- | --- |
| **Animal ID** | **Block (n)** | **Freezing (%)** | **Response area (a.u)** |  | **Animal ID** | **Block (n)** | **Freezing (%)** | **Response area (a.u)** |
| 1.00 | 1.00 | 52.43 | 60.66 |  | 1.00 | 1.00 | 52.62 | 51.66 |
| 1.00 | 2.00 | 26.14 | 20.83 |  | 1.00 | 2.00 | 46.55 | 24.11 |
| 1.00 | 3.00 | 0.00 | 44.32 |  | 1.00 | 3.00 | 2.38 | -4.30 |
| 1.00 | 4.00 | 0.00 | 12.19 |  | 1.00 | 4.00 | 15.00 | 13.96 |
| 1.00 | 5.00 | 0.00 | 27.93 |  | 1.00 | 5.00 | 0.00 | 23.35 |
| 2.00 | 1.00 | 83.52 | 24.87 |  | 2.00 | 1.00 | 81.52 | 22.01 |
| 2.00 | 2.00 | 65.52 | 20.74 |  | 2.00 | 2.00 | 45.24 | 9.48 |
| 2.00 | 3.00 | 47.34 | 18.36 |  | 2.00 | 3.00 | 76.43 | 7.19 |
| 2.00 | 4.00 | 12.39 | 4.80 |  | 2.00 | 4.00 | 49.29 | 1.62 |
| 2.00 | 5.00 | 11.76 | 6.60 |  | 2.00 | 5.00 | 47.08 | -0.71 |
| 3.00 | 1.00 | 84.62 | 9.39 |  | 3.00 | 1.00 | 81.31 | 22.15 |
| 3.00 | 2.00 | 66.07 | 9.73 |  | 3.00 | 2.00 | 44.76 | 17.61 |
| 3.00 | 3.00 | 16.82 | -3.54 |  | 3.00 | 3.00 | 30.72 | 11.38 |
| 3.00 | 4.00 | 10.37 | -10.13 |  | 3.00 | 4.00 | 21.31 | 14.09 |
| 4.00 | 1.00 | 83.81 | 8.43 |  | 3.00 | 5.00 | 10.66 | 3.37 |
| 4.00 | 2.00 | 81.55 | 14.01 |  |  |  |  |  |
| 4.00 | 3.00 | 64.05 | 6.54 |  |  |  |  |  |
| 4.00 | 4.00 | 63.08 | -12.99 |  |  |  |  |  |
| 4.00 | 5.00 | 66.36 | -13.02 |  |  |  |  |  |
| 5.00 | 1.00 | 98.57 | 16.46 |  |  |  |  |  |
| 5.00 | 2.00 | 96.07 | 6.38 |  |  |  |  |  |
| 5.00 | 3.00 | 62.62 | 0.86 |  |  |  |  |  |
| 5.00 | 4.00 | 76.43 | 6.18 |  |  |  |  |  |
| 5.00 | 5.00 | 76.49 | -1.38 |  |  |  |  |  |
| 6.00 | 1.00 | 67.38 | 0.89 |  |  |  |  |  |
| 6.00 | 2.00 | 51.07 | -1.98 |  |  |  |  |  |
| 6.00 | 3.00 | 42.74 | -9.85 |  |  |  |  |  |
| 6.00 | 4.00 | 18.45 | -2.08 |  |  |  |  |  |
| 6.00 | 5.00 | 24.45 | -7.81 |  |  |  |  |  |
| 7.00 | 1.00 | 68.21 | -12.46 |  |  |  |  |  |
| 7.00 | 2.00 | 39.05 | 7.35 |  |  |  |  |  |
| 7.00 | 3.00 | 47.14 | -21.56 |  |  |  |  |  |
| 7.00 | 4.00 | 33.09 | -2.90 |  |  |  |  |  |
| 7.00 | 5.00 | 6.11 | -13.65 |  |  |  |  |  |
