# Supplementary material for: Cerebellar modulation of memory encoding in the periaqueductal grey and fear behaviour
Source: eLife. 2022 Mar 15;11:e76278. doi: 10.7554/eLife.76278 (PMC8923669; doi:10.7554/eLife.76278)
Supplement: Figure 5—figure supplement 1—source data 1. [file elife-76278-fig5-figsupp1-data1.docx]

**Figure 5 – figure supplement 1.**

**Trial by trial comparison of conditioned behaviour in muscimol and control animals**

| **A. The percentage of time spent freezing on a trial-by-trial basis during CS+**  Individual data points showing the percentage of time spent freezing during the CS+ for each trial of extinction training (%) | | | | | | | | | | |
| --- | --- | --- | --- | --- | --- | --- | --- | --- | --- | --- |
| **Trial** | **Control Group** | | | | | | | | | |
| 1 | 80 | 25 | 65 | 70 | 90 | 90 | 65 | 85 | 65 | 100 |
| 2 | 95 | 70 | 45 | 100 | 85 | 90 | 65 | 90 | 100 | 90 |
| 3 | 95 | 90 | 85 | 75 | 100 | 100 | 40 | 95 | 60 | 100 |
| 4 | 75 | 100 | 85 | 100 | 80 | 100 | 40 | 60 | 25 | 85 |
| 5 | 50 | 100 | 80 | 100 | 100 | 100 | 55 | 60 | 95 | 55 |
| 6 | 75 | 55 | 45 | 75 | 65 | 100 | 55 | 40 | 60 | 100 |
| 7 | 80 | 80 | 40 | 85 | 90 | 100 | 55 | 85 | 100 | 90 |
| 8 | 100 | 75 | 75 | 95 | 70 | 95 | 75 | 10 | 30 | 90 |
| 9 | 85 | 100 | 75 | 90 | 100 | 100 | 60 | 50 | 70 | 65 |
| 10 | 15 | 75 | 60 | 100 | 80 | 100 | 80 | 75 | 20 | 100 |
| 11 | 20 | 100 | 60 | 100 | 100 | 100 | 60 | 80 | 75 | 65 |
| 12 | 0 | 80 | 85 | 90 | 45 | 80 | 35 | 50 | 55 | 90 |
| 13 | 0 | 85 | 70 | 35 | 100 | 60 | 90 | 70 | 90 | 65 |
| 14 | 70 | 85 | 45 | 0 | 80 | 95 | 70 | 95 | 80 | 80 |
| 15 | 0 | 65 | 50 | 55 | 100 | 75 | 60 | 25 | 90 | 65 |
| 16 | 0 | 55 | 30 | 20 | 100 | 95 | 75 | 35 | 100 | 55 |
| 17 | 0 | 100 | 40 | 45 | 70 | 55 | 65 | 0 | 75 | 85 |
| 18 | 0 | 100 | 50 | 0 | 100 | 75 | 50 | 45 | 85 | 70 |
| 19 | 0 | 80 | 5 | 70 | 70 | 85 | 30 | 20 | 75 | 80 |
| 20 | 0 | 100 | 50 | 100 | 85 | 75 | 85 | 25 | 70 | 40 |
| 21 | 0 | 75 | 75 | 0 | 70 | 30 | 60 | 30 | 10 | 60 |
| 22 | 0 | 90 | 50 | 20 | 75 | 75 | 25 | 65 | 0 | 40 |
| 23 | 0 | 80 | 40 | 0 | 75 | 90 | 0 | 55 | 65 | 10 |
| 24 | 0 | 55 | 30 | 0 | 65 | 45 | 45 | 10 | 0 | 50 |
| 25 | 0 | 45 | 0 | 0 | 45 | 65 | 0 | 55 | 80 | 60 |
| 26 | 0 | 0 | 0 | 80 | 80 | 70 | 100 | 35 | 0 | 80 |
| 27 | 0 | 0 | 0 | 0 | 60 | 70 | 40 | 40 | 0 | 90 |
| 28 | 0 | 20 | 0 | 0 | 45 | 80 | 65 | 0 | 25 | 45 |
| 29 | 0 | 0 | 0 |  | 65 | 80 | 0 | 10 | 45 | 35 |
| 30 | 0 | 0 | 35 |  | 60 | 65 | 0 | 20 | 70 | 30 |
| 31 | 0 | 0 | 0 |  | 45 | 60 | 0 | 65 | 40 | 0 |
| 32 | 0 | 0 | 0 |  | 100 | 80 | 0 | 0 | 65 | 5 |
| 33 | 0 | 0 | 0 |  | 40 | 55 | 0 | 15 | 50 | 30 |
| 34 | 0 | 0 | 0 |  | 60 | 85 | 0 | 50 | 0 | 0 |
| 35 | 0 | 0 | 0 |  | 70 | 100 | 0 | 0 | 85 | 0 |
| **Trial** | **Muscimol Group** | | | | | | | |  |  |
| 1 | 0 | 60 | 65 | 85 | 100 | 74 | 88 | 98 |  |  |
| 2 | 10 | 100 | 80 | 85 | 100 | 100 | 100 | 100 |  |  |
| 3 | 95 | 100 | 25 | 85 | 100 | 100 | 100 | 100 |  |  |
| 4 | 95 | 100 | 85 | 85 | 100 | 100 | 100 | 100 |  |  |
| 5 | 70 | 100 | 50 | 90 | 100 | 100 | 100 | 100 |  |  |
| 6 | 40 | 100 | 75 | 55 | 100 | 100 | 78 | 100 |  |  |
| 7 | 75 | 75 | 100 | 60 | 86 | 68 | 64 | 100 |  |  |
| 8 | 70 | 100 | 90 | 55 | 100 | 100 | 0 | 100 |  |  |
| 9 | 80 | 85 | 65 | 55 | 100 | 100 | 32 | 100 |  |  |
| 10 | 60 | 70 | 100 | 90 | 100 | 100 | 0 | 100 |  |  |
| 11 | 10 | 30 | 70 | 50 | 100 | 80 | 0 | 100 |  |  |
| 12 | 55 | 65 | 35 | 35 | 100 | 66 | 50 | 100 |  |  |
| 13 | 5 | 0 | 70 | 55 | 100 | 6 | 78 | 100 |  |  |
| 14 | 55 | 0 | 50 | 65 | 100 | 0 | 32 | 92 |  |  |
| 15 | 0 | 20 | 15 | 55 | 100 | 20 | 0 | 100 |  |  |
| 16 | 0 | 100 | 80 | 75 | 100 | 98 | 22 | 100 |  |  |
| 17 | 0 | 70 | 90 | 35 | 100 | 38 | 0 | 100 |  |  |
| 18 | 0 | 65 | 10 | 30 | 100 | 12 | 38 | 100 |  |  |
| 19 | 35 | 55 | 0 | 5 | 78 | 0 | 0 | 100 |  |  |
| 20 | 0 | 100 | 70 | 0 | 100 | 0 | 0 | 100 |  |  |
| 21 | 0 | 55 | 5 | 60 | 100 | 0 | 0 | 64 |  |  |
| 22 | 60 | 75 | 0 | 60 | 100 | 0 | 66 | 72 |  |  |
| 23 | 15 | 100 | 0 | 20 | 100 | 0 | 30 | 58 |  |  |
| 24 | 0 | 85 | 0 | 0 | 100 | 0 | 0 | 64 |  |  |
| 25 | 0 | 50 | 70 | 65 | 100 | 0 | 22 | 100 |  |  |
| 26 | 0 | 15 | 0 | 80 | 100 | 28 | 40 | 100 |  |  |
| 27 | 0 | 40 | 0 | 0 | 100 | 0 | 0 | 100 |  |  |
| 28 | 0 | 0 | 25 | 0 | 0 | 0 | 46 | 20 |  |  |
| 29 | 0 | 75 | 0 | 0 | 16 | 0 | 0 | 100 |  |  |
| 30 | 0 | 80 | 0 | 0 | 0 | 0 | 74 | 100 |  |  |
| 31 | 0 | 75 | 0 | 0 | 0 | 0 | 0 | 70 |  |  |
| 32 | 0 | 70 | 0 | 30 | 100 | 0 | 0 | 60 |  |  |
| 33 | 0 | 0 | 0 | 40 | 100 | 0 | 12 | 100 |  |  |
| 34 | 0 | 100 | 0 | 40 | 100 | 2 | 38 | 100 |  |  |

| **B. The percentage of time spent freezing during ITI**  Individual data points showing the percentage of time spent freezing during the ITI for each trial of extinction training (%) | | | | | | | | | | |
| --- | --- | --- | --- | --- | --- | --- | --- | --- | --- | --- |
| **Trial** | **Control Group** | | | | | | | | | |
| 1 | 71 | 74 | 85 | 100 | 88 | 92 | 37 | 65 | 60 | 100 |
| 2 | 53 | 68 | 87 | 95 | 100 | 100 | 23 | 73 | 72 | 57 |
| 3 | 58 | 84 | 77 | 100 | 100 | 100 | 37 | 63 | 83 | 70 |
| 4 | 31 | 85 | 100 | 100 | 100 | 100 | 37 | 48 | 57 | 83 |
| 5 | 66 | 98 | 87 | 56 | 87 | 100 | 42 | 43 | 72 | 28 |
| 6 | 65 | 58 | 98 | 50 | 27 | 100 | 30 | 63 | 58 | 75 |
| 7 | 23 | 87 | 49 | 91 | 85 | 98 | 63 | 42 | 70 | 64 |
| 8 | 56 | 81 | 79 | 87 | 78 | 100 | 68 | 32 | 72 | 60 |
| 9 | 52 | 84 | 73 | 100 | 75 | 100 | 63 | 48 | 57 | 45 |
| 10 | 6 | 77 | 53 | 87 | 88 | 100 | 18 | 38 | 45 | 50 |
| 11 | 23 | 97 | 65 | 92 | 95 | 97 | 10 | 65 | 53 | 33 |
| 12 | 19 | 94 | 79 | 60 | 72 | 95 | 10 | 72 | 48 | 13 |
| 13 | 18 | 89 | 65 | 11 | 75 | 97 | 35 | 72 | 65 | 37 |
| 14 | 9 | 85 | 46 | 25 | 88 | 84 | 31 | 35 | 18 | 35 |
| 15 | 0 | 58 | 58 | 21 | 100 | 82 | 63 | 23 | 37 | 45 |
| 16 | 0 | 82 | 45 | 0 | 87 | 72 | 27 | 42 | 43 | 62 |
| 17 | 0 | 69 | 48 | 53 | 55 | 92 | 32 | 28 | 58 | 70 |
| 18 | 0 | 85 | 48 | 0 | 38 | 18 | 57 | 12 | 72 | 75 |
| 19 | 0 | 74 | 52 | 34 | 50 | 87 | 57 | 10 | 47 | 20 |
| 20 | 0 | 77 | 44 | 10 | 83 | 27 | 27 | 12 | 28 | 22 |
| 21 | 0 | 52 | 36 | 0 | 35 | 62 | 7 | 8 | 14 | 37 |
| 22 | 0 | 24 | 19 | 0 | 81 | 93 | 5 | 15 | 48 | 60 |
| 23 | 0 | 23 | 19 | 0 | 73 | 60 | 0 | 43 | 32 | 65 |
| 24 | 0 | 27 | 19 | 29 | 7 | 90 | 3 | 25 | 17 | 63 |
| 25 | 0 | 5 | 0 | 0 | 40 | 75 | 7 | 42 | 23 | 8 |
| 26 | 0 | 0 | 0 | 44 | 65 | 68 | 12 | 43 | 0 | 15 |
| 27 | 0 | 50 | 0 | 0 | 89 | 68 | 22 | 23 | 5 | 17 |
| 28 | 0 | 5 | 29 | 0 | 87 | 80 | 13 | 0 | 4 | 3 |
| 29 | 0 | 0 | 0 |  | 83 | 35 | 0 | 12 | 12 | 7 |
| 30 | 0 | 0 | 32 |  | 32 | 52 | 0 | 12 | 27 | 0 |
| 31 | 0 | 0 | 0 |  | 100 | 98 | 0 | 10 | 8 | 0 |
| 32 | 0 | 0 | 0 |  | 62 | 78 | 0 | 18 | 50 | 10 |
| 33 | 0 | 0 | 0 |  | 58 | 75 | 0 | 23 | 12 | 20 |
| 34 | 0 | 0 | 37 |  | 96 | 100 | 0 | 33 | 38 | 0 |
| 35 | 0 | 0 | 8 |  | 57 | 98 | 0 | 0 | 0 | 0 |
| **Trial** | **Muscimol Group** | | | | | | | |  |  |
| 1 | 90 | 100 | 83 | 45 | 100 | 100 | 53 | 100 |  |  |
| 2 | 13 | 85 | 100 | 37 | 100 | 100 | 98 | 100 |  |  |
| 3 | 78 | 80 | 73 | 65 | 100 | 100 | 87 | 100 |  |  |
| 4 | 57 | 63 | 65 | 75 | 100 | 100 | 100 | 100 |  |  |
| 5 | 87 | 88 | 63 | 62 | 100 | 100 | 54 | 100 |  |  |
| 6 | 7 | 69 | 87 | 37 | 100 | 61 | 36 | 80 |  |  |
| 7 | 37 | 85 | 98 | 55 | 100 | 54 | 25 | 100 |  |  |
| 8 | 48 | 47 | 43 | 48 | 100 | 100 | 0 | 100 |  |  |
| 9 | 80 | 90 | 70 | 75 | 100 | 100 | 9 | 100 |  |  |
| 10 | 45 | 43 | 62 | 58 | 100 | 100 | 0 | 75 |  |  |
| 11 | 0 | 62 | 83 | 70 | 100 | 20 | 0 | 100 |  |  |
| 12 | 45 | 2 | 38 | 47 | 100 | 0 | 17 | 41 |  |  |
| 13 | 67 | 45 | 13 | 58 | 100 | 12 | 35 | 75 |  |  |
| 14 | 41 | 28 | 3 | 20 | 100 | 0 | 0 | 39 |  |  |
| 15 | 0 | 100 | 17 | 8 | 100 | 36 | 20 | 100 |  |  |
| 16 | 0 | 70 | 72 | 12 | 100 | 42 | 0 | 100 |  |  |
| 17 | 0 | 100 | 47 | 32 | 100 | 23 | 28 | 95 |  |  |
| 18 | 8 | 67 | 0 | 58 | 100 | 28 | 8 | 100 |  |  |
| 19 | 2 | 37 | 0 | 47 | 100 | 0 | 17 | 100 |  |  |
| 20 | 0 | 70 | 80 | 0 | 100 | 0 | 0 | 100 |  |  |
| 21 | 7 | 92 | 0 | 15 | 100 | 0 | 0 | 68 |  |  |
| 22 | 83 | 78 | 0 | 22 | 100 | 0 | 79 | 65 |  |  |
| 23 | 0 | 82 | 0 | 13 | 100 | 0 | 3 | 100 |  |  |
| 24 | 0 | 63 | 45 | 53 | 100 | 3 | 23 | 89 |  |  |
| 25 | 0 | 20 | 72 | 22 | 100 | 27 | 65 | 77 |  |  |
| 26 | 22 | 30 | 0 | 43 | 100 | 25 | 65 | 91 |  |  |
| 27 | 0 | 10 | 0 | 0 | 3 | 0 | 31 | 43 |  |  |
| 28 | 0 | 62 | 33 | 0 | 0 | 0 | 9 | 100 |  |  |
| 29 | 0 | 68 | 0 | 0 | 21 | 0 | 45 | 100 |  |  |
| 30 | 0 | 72 | 0 | 0 | 5 | 6 | 46 | 100 |  |  |
| 31 | 0 | 2 | 0 | 0 | 9 | 0 | 8 | 88 |  |  |
| 32 | 0 | 58 | 0 | 17 | 100 | 6 | 49 | 67 |  |  |
| 33 | 0 | 20 | 0 | 65 | 100 | 21 | 51 | 63 |  |  |
| 34 | 0 | 0 | 0 | 0 | 100 | 21 | 41 | 93 |  |  |
| 35 | 0 | 0 | 0 | 0 | 100 | 0 | 15 | 51 |  |  |

| **C. Left. The duration of freezing for each epoch**  Individual data points showing the duration of freezing epochs during baseline (B1-5), Early extinction EE (1-5) and late extinction LE(1-5). (s) | | | | | | | | | | |
| --- | --- | --- | --- | --- | --- | --- | --- | --- | --- | --- |
| **Block** | **Control Group** | | | | | | | | | |
| B1 | NF | 3 | 0.5 | 1 | 3.5 | 3.5 | NF | 0.5 | 3 | 0.5 |
| B2 | NF | 1.5 | 2.5 | 1 | 0.5 | 1 | NF | 2.5 | 5.5 | 1 |
| B3 | NF | 53.5 | 0.5 | 1 | 14.5 | 1 | NF | 1 | 1.5 | 4 |
| B4 | NF | 5 | 3.5 | 1.5 | 1 | 18.5 | NF | 1.5 | 1.5 | 7.5 |
| B5 | NF | 12 | 3 | 2.5 | 15 | 3.5 | NF | 2 | 5.5 | 1.5 |
| EE1 | 3.5 | 25.5 | 28.5 | 76.5 | 10.5 | 2 | 1.5 | 1.5 | 6.5 | 47 |
| EE2 | 2 | 7.5 | 1 | 2 | 24.5 | 6.5 | 2 | 7 | 1 | 3 |
| EE3 | 3.5 | 2.5 | 3.5 | 3.5 | 0.5 | 7.5 | 1.5 | 17 | 0.5 | 1.5 |
| EE4 | 3 | 1 | 4 | 99 | 2 | 19.5 | 2.5 | 0.5 | 2.5 | 4 |
| EE5 | 2.5 | 18.5 | 21 | 1 | 82.5 | 1.5 | 2 | 2 | 2.5 | 2.5 |
| LE1 | NF | 9.5 | 1.5 | 2 | 1 | 21 | 1 | 5.5 | 3.5 | 2 |
| LE2 | NF | 7.5 | 5.5 | 9 | 3 | 16.5 | 1 | 1 | 1 | 1 |
| LE3 | NF | 8 | 1.5 | 8 | 2.5 | 2.5 | 0.5 | 0.5 | 1 | 1 |
| LE4 | NF | 0.5 | 2.5 | 13.5 | 9 | 7.5 | 3 | 1 | 9.5 | 5 |
| LE5 | NF | 4 | 0.5 |  | 1.5 | 1.5 | 1 | 3 | 1.5 | 8 |
| **Block** | **Muscimol Group** | | | | | | | |  |  |
| B1 | NF | NF | 3 | 8.5 | 3.6 | 3.2 | 77.4 | 2.4 |  |  |
| B2 | NF | 2.5 | 1 | 1.5 | 1.4 | 2.6 | 1.4 | 7.8 |  |  |
| B3 | NF | 1 | 9.5 | 1.5 | 29 | 1 | 1.6 | 16 |  |  |
| B4 | 4.5 | 2.5 | 9 | 7 | 132 | 8.4 | 2.8 | 0.6 |  |  |
| B5 | 3 | 4 | 2 | 2 | 3.2 | 4.8 | 3.6 | 6 |  |  |
| EE1 | 28 | 60.5 | 7.5 | 9 | 192 | 185 | 22.6 | 233 |  |  |
| EE2 | 1 | 9 | 6.5 | 7 | 51.8 | 27.6 | 16.8 | 162 |  |  |
| EE3 | 3.5 | 12.5 | 13.5 | 5 | 317 | 2.2 | 32.2 | 1.4 |  |  |
| EE4 | 10 | 2 | 4 | 1 | 99 | 7.6 | 75.2 | 73.4 |  |  |
| EE5 | 32 | 31.5 | 40.5 | 14.5 | 120 | 5.6 | 2.2 | 1.8 |  |  |
| LE1 | 6.5 | 4.5 | 8.5 | 1.5 | 78.2 | 0.6 | 6.4 | 57.6 |  |  |
| LE2 | 1 | 18 | 4.5 | 7.5 | 78.2 | 1.8 | 24.2 | 19.4 |  |  |
| LE3 | 10 | 12.5 | 7.5 | 5.5 | 7.4 | 1.8 | 1 | 36.6 |  |  |
| LE4 | 15 | 29 | 12.5 | 4 | 1.6 | 5.8 | 2.4 | 5.6 |  |  |
| LE5 | 6.5 | 11 | 19 | 6.5 | 6.2 | 2 | 3.2 | 12.6 |  |  |
|  |  |  |  |  |  |  |  |  |  |  |
| NF = No freezing epochs detected | | | | | | | | | |  |
|  |  |  |  |  |  |  |  |  |  |  |
|  |  |  |  |  |  |  |  |  |  |  |
| **C. Right. Duration of movement for each epoch**  Individual data points showing the duration of movement epochs during baseline (B1-5), Early extinction EE (1-5) and late extinction LE(1-5). (s) | | | | | | | | | | |
| **Block** | **Control** | | | | | | | | | |
| B1 | 18.5 | 0.5 | 1.5 | 2 | 4.5 | 0.5 | 12 | 3 | 4 | 3.5 |
| B2 | 11.5 | 3.5 | 3.5 | 7.5 | 10 | 11 | 93.5 | 3.5 | 1 | 13 |
| B3 | 5.5 | 13.5 | 2.5 | 1 | 1.5 | 0.5 | 1 | 16.5 | 0.5 | 9 |
| B4 | 1.5 | 23.5 | 1 | 4.5 | 1 | 1 | 0.5 | 6 | 9 | 40.5 |
| B5 | 3.5 | 4.5 | 2 | 74.5 | 2 | 1 | 1 | 3 | 4 | 2.5 |
| EE1 | 1 | 12 | 2 | 1.5 | 1 | 0.5 | 1.5 | 1 | 1 | 1 |
| EE2 | 1 | 11 | 1 | 0.5 | 1 | 0.5 | 1 | 0.5 | 1.5 | 1 |
| EE3 | 1.5 | 2.5 | 1.5 | 2 | 2.5 | 1.5 | 0.5 | 6.5 | 2 | 0.5 |
| EE4 | 1.5 | 3 | 0.5 | 1 | 1.5 | 1 | 6 | 2 | 3 | 3 |
| EE5 | 2.5 | 4.5 | 3 | 2 | 1 | 1 | 0.5 | 2 | 0.5 | 3 |
| LE1 | 2 | 2.5 | 33 | 27.5 | 1 | 4 | 1.5 | 1 | 8 | 10 |
| LE2 | 3.5 | 8.5 | 1.5 | 30 | 1 | 2 | 3.5 | 6 | 11.5 | 2.5 |
| LE3 | 1.5 | 3.5 | 13 | 3.5 | 0.5 | 0.5 | 7.5 | 18 | 1 | 1 |
| LE4 | 1.5 | 2 | 1.5 | 1.5 | 1.5 | 0.5 | 62 | 2.5 | 0.5 | 2.5 |
| LE5 | 2.5 | 1.5 | 0.5 | 8 | 2 | 1 | 1 | 4 | 1 | 1 |
| **Block** | **Muscimol** | | | | | | | |  |  |
| B1 | 2 | 2 | 1 | 16.5 | 2.2 | 4.8 | 19.2 | 62.4 |  |  |
| B2 | 0.5 | 1.5 | 1 | 2 | 11 | 1 | 78.4 | 5 |  |  |
| B3 | 1.5 | 2 | 4.5 | 8.5 | 8.2 | 14.8 | 2.6 | 23.4 |  |  |
| B4 | 1.5 | 1 | 1.5 | 1 | 2.6 | 2.2 | 3.8 | 8.2 |  |  |
| B5 | 2.5 | 2 | 0.5 | 15.5 | 9.2 | 27.6 | 60 | 68.6 |  |  |
| EE1 | 3.5 | 5.5 | 3 | 0.5 | 1.6 | 1.2 | 15 | 6 |  |  |
| EE2 | 15 | 3.5 | 0.5 | 14.5 | 2.8 | 4.2 | 2.2 | 4.2 |  |  |
| EE3 | 16 | 2.5 | 2 | 1.5 | 2.8 | 1.4 | 5.6 | 3.2 |  |  |
| EE4 | 1.5 | 3.5 | 2.5 | 1.5 | 1.4 | 7.8 | 9 | 8.4 |  |  |
| EE5 | 0.5 | 2.5 | 2 | 3.5 | 1.4 | 4.8 | 3.2 | 9.4 |  |  |
| LE1 | 53.5 | 6 | 1.5 | 2.5 | 0.8 | 34.4 | 6.8 | 12.4 |  |  |
| LE2 | 1 | 2.5 | 2 | 1.5 | 0.8 | 3.4 | 7.6 | 5.2 |  |  |
| LE3 | 2 | 2.5 | 0.5 | 3.5 | 2.6 | 3.2 | 10.2 | 3.6 |  |  |
| LE4 | 2 | 2.5 | 1 | 2 | 104 | 3.2 | 52.6 | 1.6 |  |  |
| LE5 | 2 | 6.5 | 1.5 | 3.5 | 4.4 | 18.8 | 8.4 | 1.8 |  |  |
