# Supplementary material for: Cerebellar modulation of memory encoding in the periaqueductal grey and fear behaviour
Source: eLife. 2022 Mar 15;11:e76278. doi: 10.7554/eLife.76278 (PMC8923669; doi:10.7554/eLife.76278)
Supplement: Figure 5—figure supplement 2—source data 1. [file elife-76278-fig5-figsupp2-data1.docx]

**Figure 5 – figure supplement 2.**

**Conditioned fear related behaviours.**

| **A. Rate of extintion during CS+** | |  | **B. Rate of extinction during ITI** | |
| --- | --- | --- | --- | --- |
| **Control** | **Muscimol** |  | **Control** | **Muscimol** |
| -5.46 | -3.05 |  | -3.73 | -3.4 |
| 0.824 | -1.744 |  | -0.44 | -0.77 |
| -1.41 | -2.68 |  | -2.6 | -3.46 |
| -3.33 | -2.36 |  | -5.11 | -1.96 |
| -0.35 | -0.7356 |  | -1.732 | -1.4049 |
| -1.98 | -4.514 |  | -2.53 | -4.1503 |
| 0.38 | -2.7022 |  | -0.28 | -1.1837 |
| -2.85 | -1.1998 |  | -3.28 | -0.6247 |
| -0.091 |  |  | -1.9 |  |
| -1.688 |  |  | -1.85 |  |

| **C. Correlation of freezing (%) during CS+ and response area at CS+ onset for EE.** | | | | | | | | |
| --- | --- | --- | --- | --- | --- | --- | --- | --- |
|  | **Freezing (%)** | **Area** | | | | | | |
| **Control** | 59 | 7.17 |  |  |  |  |  |  |
|  | 60 | 23.97 | 12.00 | 18.56 |  |  |  |  |
|  | 52 | 20.73 | 42.70 |  |  |  |  |  |
|  | 21 | 18.86 | 15.36 | 13.74 | 14.84 | 14.77 | 11.96 |  |
|  | 21 | 3.15 | 7.68 |  |  |  |  |  |
|  | 29 | 60.87 | 9.96 |  |  |  |  |  |
|  | 50 | 4.31 | 7.90 |  |  |  |  |  |
| **Muscimol** | 43 | 53.33 | 21.84 |  |  |  |  |  |
|  | 15 | 11.74 | 21.05 | 22.94 | 12.36 | 56.93 | 64.69 | 9.84 |
|  | 58 | 12.25 |  |  |  |  |  |  |
|  |  |  |  |  |  |  |  |  |
| **D. Correlation of freezing (%) during ITI and response area at CS+ offset for EE** | | | | | | | |  |
|  | **Freezing (%)** | **Area** | | | | | |  |
| **Control** | 39 | 29.41 |  |  |  |  |  |  |
|  | 62 | 66.31 | 3.99 | 2.12 |  |  |  |  |
|  | 62 | 6.31 | 32.78 |  |  |  |  |  |
|  | 20 | 6.78 | 31.60 | 25.15 | 36.16 | 13.46 | 83.03 |  |
|  | 21 | 3.08 | 27.39 | 8.81 | 22.85 |  |  |  |
|  | 38 | 7.12 | 10.98 |  |  |  |  |  |
|  | 34 | 3.66 |  |  |  |  |  |  |
| **Muscimol** | 42 | 38.32 | 55.58 | 6.32 |  |  |  |  |
|  | 15 | 17.98 | 16.15 | 29.58 | 19.77 | 54.57 | 83.78 |  |
|  | 47 | 13.17 |  |  |  |  |  |  |

| **E. Rearing count during CS+** | | | |  | **F. Rearing count during ITI** | | | |
| --- | --- | --- | --- | --- | --- | --- | --- | --- |
| **Control EE** | **Muscimol EE** | **Control LE** | **Muscimol LE** |  | **Control EE** | **Muscimol EE** | **Control LE** | **Muscimol LE** |
| 0 | 1 | 0 | 0 |  | 0 | 4 | 1 | 5 |
| 0 | 0 | 0 | 0 |  | 0 | 0 | 0 | 0 |
| 0 | 6 | 0 | 0 |  | 0 | 9 | 0 | 5 |
| 0 | 3 | 0 | 2 |  | 2 | 10 | 1 | 5 |
| 0 | 0 | 0 | 0 |  | 0 | 0 | 0 | 0 |
| 0 | 0 | 0 | 1 |  | 0 | 0 | 0 | 0 |
| 0 | 0 | 0 | 0 |  | 0 | 0 | 6 | 0 |
| 6 | 0 | 1 | 0 |  | 26 | 0 | 17 | 0 |
| 0 |  | 0 |  |  | 0 |  | 1 |  |
| 0 |  | 0 |  |  | 0 |  | 0 |  |

| **G. USV count during CS+** | |  | **H. USV count during ITI** | |
| --- | --- | --- | --- | --- |
| **Control** | **Muscimol** |  | **Control** | **Muscimol** |
| 0 | 25 |  | 0 | 293 |
| 0 | 0 |  | 0 | 0 |
| 0 | 0 |  | 0 | 0 |
| 12 | 70 |  | 206 | 261 |
| 0 | 86 |  | 0 | 432 |
| 0 | 0 |  | 0 | 0 |
| 0 | 0 |  | 0 | 0 |
| 0 | 0 |  | 0 | 0 |
| 0 |  |  | 0 |  |
| 34 |  |  | 178 |  |
