# Supplementary material for: Cerebellar modulation of memory encoding in the periaqueductal grey and fear behaviour
Source: eLife. 2022 Mar 15;11:e76278. doi: 10.7554/eLife.76278 (PMC8923669; doi:10.7554/eLife.76278)
Supplement: Figure 6—source data 1. [file elife-76278-fig6-data1.docx]

**Figure 6.**

**MCN-vlPAG pathway inhibition and effect on behaviour during acquisition.**

| **B Top. Freezing (%) during CS-US**  Individual data points showing the percentage of time spent freezing (%) | | | | | | | | | | |
| --- | --- | --- | --- | --- | --- | --- | --- | --- | --- | --- |
| **Trial** | **Control** | | | | | | | | |  |
| 1 | 0 | 0 | 100 | 40 | 0 | 0 | 0 | 0 | 0 |  |
| 2 | 0 | 58 | 18 | 78 | 0 | 0 | 52 | 0 | 22 |  |
| 3 | 8 | 38 | 0 | 56 | 2 | 16 | 52 | 42 | 0 |  |
| 4 | 0 | 10 | 100 | 92 | 52 | 4 | 16 | 50 | 0 |  |
| 5 | 58 | 22 | 100 | 100 | 76 | 42 | 40 | 8 | 100 |  |
| 6 | 96 | 44 | 100 | 100 | 66 | 100 | 66 | 58 | 44 |  |
| 7 | 100 | 96 | 100 | 100 | 56 | 100 | 74 | 100 | 72 |  |
| **Trial** | **DREADD** | | | | | | | | | |
| 1 | 0 | 0 | 0 | 0 | 0 | 0 | 0 | 0 | 0 | 14 |
| 2 | 8 | 84 | 0 | 10 | 44 | 38 | 8 | 0 | 38 | 66 |
| 3 | 78 | 56 | 0 | 26 | 40 | 40 | 88 | 0 | 40 | 74 |
| 4 | 44 | 72 | 0 | 26 | 38 | 42 | 28 | 42 | 100 | 52 |
| 5 | 48 | 20 | 0 | 74 | 34 | 72 | 100 | 0 | 100 | 100 |
| 6 | 98 | 100 | 0 | 30 | 0 | 18 | 100 | 82 | 30 | 100 |
| 7 | 100 | 100 | 0 | 0 | 58 | 100 | 96 | 100 | 100 | 100 |
|  |  |  |  |  |  |  |  |  |  |  |
| **B Bottom. Freezing (%) during ITI**  Individual data points showing the percentage of time spent freezing (%) | | | | | | | | | | |
| **Trial** | **Control** | | | | | | | | |  |
| 1 | 0 | 33 | 61 | 51 | 0 | 0 | 5 | 0 | 0 |  |
| 2 | 37 | 79 | 51 | 72 | 45 | 42 | 41 | 9 | 25 |  |
| 3 | 0 | 75 | 45 | 67 | 41 | 17 | 69 | 39 | 8 |  |
| 4 | 15 | 90 | 93 | 85 | 82 | 55 | 40 | 42 | 20 |  |
| 5 | 37 | 33 | 92 | 83 | 58 | 9 | 51 | 65 | 49 |  |
| 6 | 90 | 73 | 95 | 71 | 82 | 75 | 76 | 72 | 43 |  |
| 7 | 84 | 68 | 100 | 71 | 65 | 71 | 65 | 73 | 0 |  |
| **Trial** | **DREADD** | | | | | | | | | |
| 1 | 22 | 11 | 0 | 0 | 6 | 27 | 2 | 0 | 10 | 14 |
| 2 | 25 | 76 | 0 | 13 | 10 | 29 | 26 | 3 | 19 | 43 |
| 3 | 20 | 75 | 0 | 22 | 41 | 45 | 24 | 4 | 67 | 52 |
| 4 | 1 | 15 | 0 | 34 | 0 | 55 | 51 | 0 | 54 | 91 |
| 5 | 53 | 79 | 0 | 31 | 27 | 44 | 43 | 41 | 29 | 91 |
| 6 | 70 | 92 | 0 | 0 | 53 | 63 | 53 | 63 | 55 | 93 |
| 7 | 93 | 95 | 0 | 14 | 73 | 55 | 62 | 53 | 75 | 94 |

| **C Top. USV count during CS-US**  Individual data points showing total number of USVs (n) | |  | **C Bottom. USV count during ITI**  Individual data points showing total number of USVs (n) | |
| --- | --- | --- | --- | --- |
| **Control** | **DREADD** |  | **Control** | **DREADD** |
| 4 | 0 |  | 84 | 0 |
| 16 | 6 |  | 189 | 78 |
| 1 | 0 |  | 40 | 0 |
| 31 | 0 |  | 205 | 0 |
| 0 | 0 |  | 13 | 0 |
| 0 | 0 |  | 24 | 0 |
| 0 | 0 |  | 3 | 0 |
| 0 | 0 |  | 0 | 0 |
| 0 | 4 |  | 0 | 4 |
|  | 17 |  |  | 85 |
