# Supplementary material for: Cerebellar modulation of memory encoding in the periaqueductal grey and fear behaviour
Source: eLife. 2022 Mar 15;11:e76278. doi: 10.7554/eLife.76278 (PMC8923669; doi:10.7554/eLife.76278)
Supplement: Figure 6—figure supplement 2—source data 1. [file elife-76278-fig6-figsupp2-data1.docx]

**Figure 6 – figure supplement 2.**

**Effect of DREADDs on general motor and affective behaviour.**

| **B. Percentage of time spent in the open or closed arm of an elevated plus maze**  Individual data points showing time spent in either the open or closed arms (%) | | | |
| --- | --- | --- | --- |
| **Open** | | **Closed** | |
| **Control** | **DREADD** | **Control** | **DREADD** |
| 3.68 | 12.39 | 96.32 | 87.61 |
| 12.90 | 33.27 | 87.10 | 66.73 |
| 24.97 | 0.00 | 75.03 | 100.00 |
| 6.52 | 5.75 | 93.48 | 94.25 |
| 12.16 | 27.55 | 87.84 | 72.45 |
| 0.00 | 10.52 | 100.00 | 89.48 |
| 18.26 | 22.77 | 81.74 | 77.23 |
| 21.29 | 16.32 | 78.71 | 83.68 |
| 7.87 | 21.03 | 92.13 | 78.97 |
| **C. Time spent in central versus peripheral regions of the open field maze**  Individual data points showing time spent in open field (s) | | | |
| **Centre** | | **Periphery** | |
| **Control** | **DREADD** | **Control** | **DREADD** |
| 69.12 | 14.72 | 520.64 | 585.28 |
| 35.97 | 39.83 | 536.85 | 560.17 |
| 16.48 | 15.45 | 583.52 | 550.57 |
| 0.33 | 17.44 | 567.08 | 582.56 |
| 11.06 | 17.17 | 582.03 | 582.69 |
| 15.25 | 17.31 | 584.75 | 582.69 |
| 10.60 |  | 588.44 |  |
| 14.78 |  | 584.92 |  |

| **D. Distance travelled in an open field maze**  Individual points showing total distance travelled (cm) | |
| --- | --- |
| **Control** | **DREADD** |
| 58.65 | 36.36 |
| 37.35 | 35.24 |
| 24.25 | 36.57 |
| 28.22 | 48.10 |
| 33.59 | 44.63 |
| 44.82 | 37.62 |
| 32.98 |  |
| 47.47 |  |

| **E. Beam balance performance**  Individual points showing average time to traverse the beam (s) | | | |
| --- | --- | --- | --- |
| **Control** | | **DREADD** | |
| **Baseline** | **CNO** | **Baseline** | **CNO** |
| 5.4 | 4.2 | 18.3 | 16.4 |
| 5.7 | 3.9 | 6.6 | 4.6 |
| 15.1 | 20.5 | 4.9 | 4.0 |
| 5.5 | 3.7 | 4.9 | 4.3 |
| 4.9 | 4.9 | 5.2 | 3.8 |
| 7.7 | 6.2 | 18.7 | 14.8 |
| 4.2 | 4.4 | 7.7 | 6.8 |
| 5.1 | 4.5 | 8.4 | 8.7 |
|  | 4.8 | 5.8 | 4.7 |

| **F. Beam balance performance after i.p. CNO**  Individual points showing average time to traverse the beam (s) | | | |
| --- | --- | --- | --- |
| **Control** | | **DREADD** | |
| **Baseline** | **CNO** | **Baseline** | **CNO** |
| 4.1 | 4.3 | 7.9 | 13.3 |
| 5.4 | 6.1 | 4.0 | 7.5 |
| 13.5 | 4.8 | 16.8 | 12.0 |
| 5.4 | 3.8 | 4.2 | 7.6 |
| 3.3 | 5.6 | 6.6 | 11.4 |
| 4.4 | 5.8 | 6.5 | 5.9 |
| 4.3 | 5.6 | 9.5 | 9.7 |
| 4.4 | 3.3 | 6.4 | 11.4 |
| 4.4 | 3.9 | 4.6 | 28.2 |
