# Supplementary material for: Cerebellar modulation of memory encoding in the periaqueductal grey and fear behaviour
Source: eLife. 2022 Mar 15;11:e76278. doi: 10.7554/eLife.76278 (PMC8923669; doi:10.7554/eLife.76278)
Supplement: Figure 6—figure supplement 3—source data 1. [file elife-76278-fig6-figsupp3-data1.docx]

**Figure 6 – figure supplement 3.**

**Anatomical and physiological evaluation of DREADD transfection of MCN-PAG pathway.**

| **C. Estimation of double labelled neurons**  Percentage of double labelled neurons (%) per animal | |
| --- | --- |
| **overlap/antero** | **overlap/retro** |
| 23.12 | 83.33 |
| 48.63 | 51.80 |
| 31.96 | 51.45 |
| 9.08 | 89.67 |
