# Supplementary material for: Cerebellar modulation of memory encoding in the periaqueductal grey and fear behaviour
Source: eLife. 2022 Mar 15;11:e76278. doi: 10.7554/eLife.76278 (PMC8923669; doi:10.7554/eLife.76278)
Supplement: Figure 7—source data 1. [file elife-76278-fig7-data1.docx]

**Figure 7.**

**MCN-vPAG pathway inhibition effect on behaviour during extinction.**

| **A. Freezing (%) during CS+**  Individual data points showing the percentage of time animals displayed freezing behaviour (%) | | | |  | **B. Freezing (%) during ITI**  Individual data points showing the percentage of time animals displayed freezing behaviour (%) | | | |
| --- | --- | --- | --- | --- | --- | --- | --- | --- |
| **Control** | | **DREADD** | |  | **Control** | | **DREADD** | |
| **EE** | **LE** | **EE** | **LE** |  | **EE** | **LE** | **EE** | **LE** |
| 100 | 58 | 88 | 48 |  | 100 | 64 | 90 | 59 |
| 90 | 92 | 81 | 29 |  | 99 | 90 | 85 | 26 |
| 95 | 5 | 91 | 8 |  | 99 | 5 | 98 | 5 |
| 89 | 30 | 84 | 44 |  | 96 | 32 | 62 | 36 |
| 52 | 14 | 74 | 34 |  | 57 | 25 | 84 | 18 |
| 61 | 67 | 68 | 2 |  | 65 | 54 | 71 | 9 |
| 99 | 37 | 74 | 37 |  | 94 | 41 | 94 | 41 |
| 60 | 41 | 62 | 9 |  | 53 | 21 | 62 | 7 |
| 28 | 12 | 69 | 34 |  | 21 | 14 | 82 | 45 |
|  |  | 98 | 49 |  |  |  | 99 | 51 |

| **C. Rate of extinction during CS+**  Individual data points showing rate of extinction per animal (%/trial) | |  | **D. Rate of extinction during ITI**  Individual data points showing rate of extinction per animal (%/trial) | |
| --- | --- | --- | --- | --- |
| **Control** | **DREADD** |  | **Control** | **DREADD** |
| -1.6338 | -1.3117 |  | -1.2173 | -2.168 |
| 1.51 | 0.65 |  | 0.19 | 1.91 |
| -5.28 | -2.14 |  | -4.98 | -3.35 |
| -4.72 | -1.02 |  | -3.36 | -1.38 |
| -3.95 | 0.37 |  | -3.46 | -1.39 |
| -4.58 | -2.04 |  | -3.87 | -2.08 |
| -3.64 | -2.05 |  | 0.09 | 0.09 |
| 0.20 | -2.00 |  | -1.77 | -3.36 |
| -2.89 | -0.15 |  | -1.80 | -1.52 |
|  | 0.15 |  |  | -0.71 |

| **E. Rearing count during CS+**  Individual data points showing total rearing count per animal (n) | | | |  | **F. Rearing count during ITI**  Individual data points showing total rearing count per animal (n) | | | |
| --- | --- | --- | --- | --- | --- | --- | --- | --- |
| **Control** | | **DREADD** | |  | **Control** | | **DREADD** | |
| **EE** | **LE** | **EE** | **LE** |  | **EE** | **LE** | **EE** | **LE** |
| 0.00 | 1.00 | 0.00 | 0.00 |  | 0.00 | 1.00 | 0.00 | 2.00 |
| 0.00 | 0.00 | 0.00 | 0.00 |  | 0.00 | 0.00 | 2.00 | 1.00 |
| 0.00 | 1.00 | 0.00 | 0.00 |  | 0.00 | 3.00 | 0.00 | 2.00 |
| 0.00 | 2.00 | 0.00 | 0.00 |  | 0.00 | 1.00 | 2.00 | 3.00 |
| 0.00 | 3.00 | 0.00 | 0.00 |  | 0.00 | 8.00 | 0.00 | 2.00 |
| 0.00 | 2.00 | 0.00 | 0.00 |  | 0.00 | 2.00 | 2.00 | 0.00 |
| 0.00 | 1.00 | 0.00 | 0.00 |  | 0.00 | 1.00 | 0.00 | 1.00 |
| 1.00 | 0.00 | 0.00 | 1.00 |  | 3.00 | 6.00 | 4.00 | 10.00 |
| 0.00 | 0.00 | 0.00 | 4.00 |  | 7.00 | 1.00 | 0.00 | 1.00 |
|  |  | 0.00 | 0.00 |  |  |  | 0.00 | 0.00 |

| **G. USV count during CS+**  Individual data points showing total number of USVs per animal (n) | |  | **H. USV count during ITI**  Individual data points showing total number of USVs per animal (n) | |
| --- | --- | --- | --- | --- |
| **Control** | **DREADD** |  | **Control** | **DREADD** |
| 0.00 | 0.00 |  | 0.00 | 19.00 |
| 49.00 | 0.00 |  | 379.00 | 0.00 |
| 3.00 | 0.00 |  | 19.00 | 0.00 |
| 6.00 | 0.00 |  | 116.00 | 0.00 |
| 0.00 | 1.00 |  | 0.00 | 46.00 |
| 0.00 | 21.00 |  | 0.00 | 89.00 |
| 0.00 | 0.00 |  | 30.00 | 0.00 |
| 0.00 | 0.00 |  | 0.00 | 6.00 |
| 0.00 | 6.00 |  | 0.00 | 5.00 |
|  | 20.00 |  |  | 139.00 |
