# Supplementary material for: Cerebellar modulation of memory encoding in the periaqueductal grey and fear behaviour
Source: eLife. 2022 Mar 15;11:e76278. doi: 10.7554/eLife.76278 (PMC8923669; doi:10.7554/eLife.76278)
Supplement: Figure 7—figure supplement 1—source data 1. [file elife-76278-fig7-figsupp1-data1.docx]

**Figure 7 – figure supplement 1.**

**Freezing and USV behaviour across extinction training.**

| **A. Trial by trial freezing behaviour during CS+**  Individual data points showing the percentage of time spent freezing during each trial of extinction training (%) | | | | | | | | | |  |
| --- | --- | --- | --- | --- | --- | --- | --- | --- | --- | --- |
| **Trial** | **Control** | | | | | | | | |  |
| 1 | 100 | 8 | 88 | 100 | 96 | 44 | 100 | 46 | 54 |  |
| 2 | 100 | 56 | 100 | 100 | 36 | 100 | 100 | 30 | 58 |  |
| 3 | 100 | 100 | 100 | 100 | 94 | 100 | 100 | 74 | 68 |  |
| 4 | 100 | 100 | 100 | 100 | 56 | 48 | 100 | 100 | 100 |  |
| 5 | 100 | 100 | 100 | 100 | 92 | 100 | 100 | 58 | 38 |  |
| 6 | 100 | 100 | 100 | 100 | 84 | 74 | 100 | 100 | 74 |  |
| 7 | 100 | 100 | 100 | 100 | 74 | 94 | 100 | 100 | 0 |  |
| 8 | 100 | 100 | 70 | 86 | 52 | 80 | 82 | 70 | 0 |  |
| 9 | 100 | 100 | 66 | 86 | 16 | 100 | 100 | 6 | 0 |  |
| 10 | 100 | 100 | 100 | 100 | 0 | 78 | 100 | 0 | 0 |  |
| 11 | 100 | 100 | 100 | 100 | 38 | 30 | 100 | 62 | 0 |  |
| 12 | 100 | 100 | 100 | 48 | 22 | 0 | 100 | 66 | 0 |  |
| 13 | 100 | 100 | 100 | 50 | 20 | 0 | 100 | 62 | 0 |  |
| 14 | 100 | 100 | 100 | 78 | 44 | 0 | 100 | 66 | 0 |  |
| 15 | 100 | 100 | 0 | 0 | 0 | 38 | 100 | 74 | 50 |  |
| 16 | 100 | 100 | 100 | 88 | 0 | 30 | 100 | 78 | 0 |  |
| 17 | 100 | 74 | 10 | 0 | 0 | 18 | 50 | 56 | 80 |  |
| 18 | 76 | 100 | 0 | 28 | 0 | 10 | 100 | 82 | 0 |  |
| 19 | 84 | 100 | 0 | 32 | 0 | 42 | 0 | 68 | 0 |  |
| 20 | 62 | 100 | 0 | 58 | 50 | 0 | 16 | 60 | 0 |  |
| 21 | 38 | 100 | 0 | 0 | 20 | 0 | 0 | 58 | 0 |  |
| 22 | 44 | 100 | 0 | 4 | 60 | 28 | 14 | 60 | 0 |  |
| 23 | 100 | 100 | 74 | 0 | 76 | 48 | 22 | 60 | 0 |  |
| 24 | 100 | 100 | 100 | 40 | 68 | 0 | 8 | 30 | 0 |  |
| 25 | 88 | 80 | 0 | 86 | 42 | 0 | 100 | 82 | 0 |  |
| 26 | 100 | 100 | 0 | 68 | 20 | 40 | 70 | 70 | 0 |  |
| 27 | 100 | 100 | 10 | 90 | 0 | 32 | 48 | 80 | 0 |  |
| 28 | 100 | 100 | 0 | 56 | 14 | 0 | 76 | 0 | 0 |  |
| 29 | 52 | 82 | 0 | 74 | 14 | 0 | 4 | 64 | 0 |  |
| 30 | 16 | 76 | 0 | 56 | 84 | 0 | 58 | 0 | 32 |  |
| 31 | 0 | 100 | 0 | 56 | 32 | 34 | 62 | 30 | 0 |  |
| 32 | 74 | 100 | 0 | 72 | 42 | 74 | 52 | 58 | 28 |  |
| 33 | 12 | 100 | 0 | 24 | 0 | 54 | 58 | 76 | 6 |  |
| 34 | 100 | 100 | 0 | 42 | 34 | 48 | 44 | 18 | 0 |  |
| 35 | 0 | 66 | 40 | 22 | 0 | 58 | 28 | 74 | 0 |  |
| **Trial** | **DREADDs** | | | | | | | | | |
| 1 | 54 | 100 | 16 | 76 | 48 | 38 | 56 | 78 | 0 | 68 |
| 2 | 100 | 100 | 94 | 100 | 100 | 100 | 100 | 100 | 100 | 100 |
| 3 | 70 | 0 | 92 | 100 | 100 | 52 | 100 | 100 | 100 | 100 |
| 4 | 100 | 10 | 100 | 56 | 100 | 100 | 100 | 76 | 100 | 100 |
| 5 | 100 | 100 | 100 | 100 | 100 | 74 | 100 | 28 | 100 | 100 |
| 6 | 100 | 100 | 100 | 74 | 100 | 100 | 58 | 100 | 10 | 100 |
| 7 | 100 | 100 | 100 | 100 | 100 | 72 | 100 | 66 | 100 | 100 |
| 8 | 100 | 100 | 100 | 94 | 16 | 100 | 18 | 28 | 100 | 100 |
| 9 | 100 | 100 | 100 | 76 | 40 | 62 | 58 | 100 | 100 | 100 |
| 10 | 100 | 100 | 100 | 90 | 36 | 68 | 18 | 0 | 100 | 100 |
| 11 | 100 | 100 | 100 | 100 | 100 | 48 | 70 | 18 | 100 | 100 |
| 12 | 100 | 100 | 100 | 74 | 100 | 0 | 74 | 62 | 0 | 100 |
| 13 | 100 | 52 | 78 | 88 | 100 | 44 | 92 | 32 | 60 | 100 |
| 14 | 12 | 78 | 100 | 46 | 0 | 90 | 90 | 76 | 0 | 100 |
| 15 | 60 | 66 | 22 | 60 | 100 | 0 | 58 | 0 | 100 | 48 |
| 16 | 100 | 82 | 26 | 100 | 100 | 30 | 74 | 40 | 20 | 100 |
| 17 | 74 | 100 | 100 | 72 | 38 | 30 | 44 | 4 | 100 | 100 |
| 18 | 26 | 100 | 76 | 60 | 100 | 10 | 46 | 0 | 62 | 100 |
| 19 | 46 | 100 | 82 | 86 | 100 | 44 | 26 | 64 | 100 | 100 |
| 20 | 100 | 100 | 10 | 86 | 100 | 78 | 42 | 100 | 100 | 100 |
| 21 | 82 | 46 | 24 | 52 | 100 | 74 | 56 | 70 | 58 | 100 |
| 22 | 30 | 0 | 0 | 82 | 2 | 0 | 38 | 0 | 0 | 0 |
| 23 | 74 | 0 | 0 | 10 | 74 | 100 | 100 | 32 | 0 | 100 |
| 24 | 76 | 38 | 0 | 18 | 0 | 84 | 14 | 90 | 26 | 18 |
| 25 | 52 | 10 | 0 | 48 | 100 | 100 | 46 | 16 | 72 | 100 |
| 26 | 100 | 88 | 66 | 76 | 32 | 34 | 100 | 30 | 20 | 100 |
| 27 | 24 | 0 | 94 | 68 | 32 | 24 | 14 | 62 | 0 | 100 |
| 28 | 100 | 0 | 78 | 12 | 42 | 50 | 0 | 28 | 0 | 100 |
| 29 | 90 | 0 | 82 | 0 | 0 | 54 | 0 | 0 | 18 | 100 |
| 30 | 100 | 8 | 100 | 64 | 18 | 50 | 0 | 68 | 80 | 100 |
| 31 | 100 | 48 | 30 | 100 | 18 | 0 | 100 | 0 | 28 | 100 |
| 32 | 100 | 50 | 92 | 32 | 56 | 30 | 68 | 0 | 18 | 100 |
| 33 | 100 | 100 | 32 | 60 | 14 | 14 | 42 | 32 | 0 | 100 |
| 34 | 66 | 0 | 80 | 20 | 28 | 84 | 16 | 42 | 14 | 100 |
| 35 | 82 | 56 | 34 | 70 | 32 | 0 | 46 | 0 | 8 | 100 |

| **B. Trial by trial freezing behaviour during ITI**  Individual data points showing the percentage of time spent freezing during each trial of extinction training (%) | | | | | | | | | |  |
| --- | --- | --- | --- | --- | --- | --- | --- | --- | --- | --- |
| **Trial** | **Control** | | | | | | | | |  |
| 1 | 100 | 85 | 100 | 100 | 60 | 82 | 100 | 67 | 54 |  |
| 2 | 100 | 100 | 100 | 100 | 96 | 67 | 100 | 30 | 59 |  |
| 3 | 100 | 100 | 100 | 100 | 74 | 74 | 100 | 87 | 60 |  |
| 4 | 100 | 100 | 100 | 100 | 81 | 73 | 100 | 65 | 31 |  |
| 5 | 100 | 100 | 100 | 100 | 95 | 92 | 100 | 100 | 63 |  |
| 6 | 100 | 100 | 100 | 100 | 87 | 60 | 100 | 89 | 3 |  |
| 7 | 100 | 100 | 100 | 100 | 79 | 83 | 67 | 100 | 0 |  |
| 8 | 100 | 100 | 98 | 99 | 17 | 71 | 68 | 65 | 8 |  |
| 9 | 100 | 100 | 100 | 100 | 0 | 84 | 97 | 24 | 6 |  |
| 10 | 100 | 100 | 97 | 100 | 19 | 83 | 83 | 14 | 0 |  |
| 11 | 100 | 100 | 94 | 87 | 39 | 17 | 100 | 9 | 0 |  |
| 12 | 100 | 100 | 97 | 94 | 31 | 11 | 100 | 30 | 17 |  |
| 13 | 100 | 100 | 100 | 72 | 61 | 50 | 100 | 19 | 0 |  |
| 14 | 100 | 100 | 94 | 93 | 53 | 61 | 100 | 44 | 0 |  |
| 15 | 100 | 100 | 57 | 88 | 0 | 16 | 100 | 15 | 12 |  |
| 16 | 100 | 100 | 15 | 56 | 14 | 19 | 100 | 31 | 28 |  |
| 17 | 100 | 100 | 27 | 15 | 0 | 30 | 91 | 48 | 49 |  |
| 18 | 95 | 100 | 43 | 72 | 23 | 7 | 91 | 55 | 0 |  |
| 19 | 97 | 100 | 0 | 77 | 24 | 0 | 92 | 49 | 23 |  |
| 20 | 85 | 100 | 0 | 49 | 35 | 35 | 100 | 33 | 0 |  |
| 21 | 26 | 100 | 31 | 11 | 29 | 20 | 100 | 67 | 13 |  |
| 22 | 92 | 95 | 18 | 0 | 17 | 25 | 100 | 29 | 0 |  |
| 23 | 96 | 100 | 23 | 13 | 44 | 22 | 84 | 50 | 0 |  |
| 24 | 100 | 53 | 5 | 37 | 15 | 0 | 81 | 71 | 0 |  |
| 25 | 100 | 82 | 0 | 67 | 19 | 0 | 100 | 81 | 0 |  |
| 26 | 100 | 91 | 0 | 73 | 0 | 0 | 55 | 69 | 3 |  |
| 27 | 100 | 86 | 13 | 81 | 13 | 0 | 0 | 27 | 19 |  |
| 28 | 89 | 100 | 55 | 100 | 16 | 0 | 0 | 27 | 5 |  |
| 29 | 21 | 100 | 0 | 37 | 40 | 0 | 92 | 24 | 35 |  |
| 30 | 32 | 100 | 0 | 71 | 21 | 12 | 77 | 10 | 9 |  |
| 31 | 5 | 100 | 0 | 49 | 14 | 76 | 100 | 33 | 21 |  |
| 32 | 44 | 100 | 0 | 64 | 18 | 52 | 96 | 45 | 0 |  |
| 33 | 73 | 100 | 0 | 67 | 11 | 48 | 93 | 48 | 0 |  |
| 34 | 49 | 95 | 7 | 63 | 44 | 62 | 100 | 24 | 10 |  |
| 35 | 16 | 94 | 29 | 55 | 13 | 19 | 94 | 46 | 6 |  |
| **Trial** | **DREADDs** | | | | | | | | | |
| 1 | 88 | 100 | 97 | 100 | 100 | 69 | 100 | 87 | 93 | 100 |
| 2 | 99 | 8 | 90 | 100 | 100 | 71 | 100 | 91 | 100 | 100 |
| 3 | 89 | 0 | 89 | 64 | 100 | 55 | 100 | 82 | 100 | 100 |
| 4 | 100 | 100 | 100 | 85 | 100 | 95 | 100 | 35 | 100 | 100 |
| 5 | 100 | 100 | 100 | 16 | 100 | 95 | 100 | 100 | 94 | 100 |
| 6 | 100 | 100 | 100 | 37 | 100 | 100 | 100 | 100 | 56 | 100 |
| 7 | 100 | 100 | 100 | 49 | 63 | 81 | 67 | 55 | 100 | 100 |
| 8 | 100 | 100 | 100 | 42 | 38 | 78 | 68 | 93 | 99 | 100 |
| 9 | 100 | 100 | 100 | 19 | 53 | 79 | 97 | 26 | 100 | 100 |
| 10 | 100 | 97 | 100 | 100 | 100 | 80 | 83 | 69 | 100 | 100 |
| 11 | 100 | 100 | 100 | 79 | 85 | 65 | 100 | 9 | 82 | 100 |
| 12 | 100 | 92 | 100 | 63 | 55 | 35 | 100 | 35 | 59 | 89 |
| 13 | 61 | 100 | 100 | 84 | 81 | 48 | 100 | 45 | 65 | 100 |
| 14 | 28 | 100 | 93 | 26 | 98 | 51 | 100 | 49 | 0 | 100 |
| 15 | 95 | 100 | 1 | 22 | 100 | 25 | 100 | 0 | 37 | 33 |
| 16 | 70 | 100 | 32 | 35 | 98 | 62 | 100 | 22 | 100 | 100 |
| 17 | 35 | 100 | 40 | 36 | 94 | 25 | 91 | 13 | 62 | 100 |
| 18 | 50 | 100 | 61 | 73 | 100 | 55 | 91 | 35 | 100 | 100 |
| 19 | 96 | 100 | 47 | 33 | 77 | 28 | 92 | 92 | 100 | 100 |
| 20 | 57 | 100 | 39 | 69 | 39 | 71 | 100 | 12 | 75 | 100 |
| 21 | 75 | 85 | 57 | 51 | 36 | 59 | 100 | 17 | 62 | 73 |
| 22 | 43 | 9 | 19 | 17 | 45 | 75 | 100 | 0 | 0 | 41 |
| 23 | 87 | 10 | 0 | 39 | 23 | 48 | 84 | 26 | 37 | 67 |
| 24 | 89 | 12 | 0 | 61 | 67 | 61 | 81 | 19 | 89 | 100 |
| 25 | 78 | 16 | 0 | 49 | 46 | 40 | 100 | 22 | 29 | 100 |
| 26 | 69 | 20 | 59 | 15 | 15 | 23 | 55 | 25 | 0 | 100 |
| 27 | 76 | 7 | 100 | 36 | 31 | 40 | 0 | 41 | 14 | 100 |
| 28 | 92 | 0 | 42 | 71 | 9 | 28 | 0 | 27 | 0 | 100 |
| 29 | 88 | 0 | 46 | 13 | 2 | 25 | 92 | 0 | 17 | 100 |
| 30 | 67 | 45 | 66 | 87 | 51 | 31 | 77 | 15 | 75 | 54 |
| 31 | 100 | 77 | 71 | 77 | 23 | 7 | 100 | 11 | 71 | 89 |
| 32 | 80 | 93 | 36 | 59 | 29 | 67 | 96 | 16 | 12 | 89 |
| 33 | 75 | 9 | 33 | 25 | 31 | 20 | 93 | 59 | 0 | 65 |
| 34 | 71 | 11 | 34 | 89 | 31 | 24 | 100 | 10 | 64 | 65 |
| 35 | 43 | 39 | 11 | 3 | 10 | 35 | 94 | 0 | 42 | 100 |

| **C. Trial by trial USVs emission during CS+**  Individual data points showing the USV emitted during each trial of extinction training (%) | | | | | | | | | |  |
| --- | --- | --- | --- | --- | --- | --- | --- | --- | --- | --- |
| **Trial** | **Control Group** | | | | | | | | |  |
| 1 | 0 | 0 | 0 | 0 | 0 | 0 | 0 | 0 | 0 |  |
| 2 | 0 | 0 | 0 | 0 | 0 | 0 | 0 | 0 | 0 |  |
| 3 | 0 | 0 | 0 | 0 | 0 | 0 | 0 | 0 | 0 |  |
| 4 | 0 | 0 | 0 | 0 | 0 | 0 | 0 | 0 | 0 |  |
| 5 | 0 | 0 | 0 | 1 | 0 | 0 | 0 | 0 | 0 |  |
| 6 | 0 | 0 | 0 | 1 | 0 | 0 | 0 | 0 | 0 |  |
| 7 | 0 | 0 | 0 | 2 | 0 | 0 | 0 | 0 | 0 |  |
| 8 | 0 | 0 | 0 | 0 | 0 | 0 | 0 | 0 | 0 |  |
| 9 | 0 | 2 | 0 | 0 | 0 | 0 | 0 | 0 | 0 |  |
| 10 | 0 | 2 | 0 | 1 | 0 | 0 | 0 | 0 | 0 |  |
| 11 | 0 | 1 | 0 | 0 | 0 | 0 | 0 | 0 | 0 |  |
| 12 | 0 | 1 | 0 | 0 | 0 | 0 | 0 | 0 | 0 |  |
| 13 | 0 | 3 | 1 | 0 | 0 | 0 | 0 | 0 | 0 |  |
| 14 | 0 | 2 | 2 | 1 | 0 | 0 | 0 | 0 | 0 |  |
| 15 | 0 | 2 | 0 | 0 | 0 | 0 | 0 | 0 | 0 |  |
| 16 | 0 | 1 | 0 | 0 | 0 | 0 | 0 | 0 | 0 |  |
| 17 | 0 | 4 | 0 | 0 | 0 | 0 | 0 | 0 | 0 |  |
| 18 | 0 | 2 | 0 | 0 | 0 | 0 | 0 | 0 | 0 |  |
| 19 | 0 | 2 | 0 | 0 | 0 | 0 | 0 | 0 | 0 |  |
| 20 | 0 | 3 | 0 | 0 | 0 | 0 | 0 | 0 | 0 |  |
| 21 | 0 | 3 | 0 | 0 | 0 | 0 | 0 | 0 | 0 |  |
| 22 | 0 | 3 | 0 | 0 | 0 | 0 | 0 | 0 | 0 |  |
| 23 | 0 | 1 | 0 | 0 | 0 | 0 | 0 | 0 | 0 |  |
| 24 | 0 | 3 | 0 | 0 | 0 | 0 | 0 | 0 | 0 |  |
| 25 | 0 | 0 | 0 | 0 | 0 | 0 | 0 | 0 | 0 |  |
| 26 | 0 | 2 | 0 | 0 | 0 | 0 | 0 | 0 | 0 |  |
| 27 | 0 | 3 | 0 | 0 | 0 | 0 | 0 | 0 | 0 |  |
| 28 | 0 | 3 | 0 | 0 | 0 | 0 | 0 | 0 | 0 |  |
| 29 | 0 | 0 | 0 | 0 | 0 | 0 | 0 | 0 | 0 |  |
| 30 | 0 | 0 | 0 | 0 | 0 | 0 | 0 | 0 | 0 |  |
| 31 | 0 | 1 | 0 | 0 | 0 | 0 | 0 | 0 | 0 |  |
| 32 | 0 | 2 | 0 | 0 | 0 | 0 | 0 | 0 | 0 |  |
| 33 | 0 | 1 | 0 | 0 | 0 | 0 | 0 | 0 | 0 |  |
| 34 | 0 | 1 | 0 | 0 | 0 | 0 | 0 | 0 | 0 |  |
| 35 | 0 | 0 | 0 | 0 | 0 | 0 | 0 | 0 | 0 |  |
| **Trial** | **DREADD group** | | | | | | | | | |
| 1 | 0 | 0 | 0 | 0 | 0 | 0 | 0 | 0 | 0 | 0 |
| 2 | 0 | 0 | 0 | 0 | 0 | 0 | 0 | 0 | 0 | 3 |
| 3 | 0 | 0 | 0 | 0 | 0 | 0 | 0 | 0 | 0 | 1 |
| 4 | 0 | 0 | 0 | 0 | 0 | 0 | 0 | 0 | 0 | 1 |
| 5 | 0 | 0 | 0 | 0 | 0 | 0 | 0 | 0 | 0 | 1 |
| 6 | 0 | 0 | 0 | 0 | 0 | 0 | 0 | 0 | 0 | 3 |
| 7 | 0 | 0 | 0 | 0 | 0 | 0 | 0 | 0 | 0 | 1 |
| 8 | 0 | 0 | 0 | 0 | 0 | 0 | 0 | 0 | 0 | 1 |
| 9 | 0 | 0 | 0 | 0 | 0 | 0 | 0 | 0 | 0 | 1 |
| 10 | 0 | 0 | 0 | 0 | 0 | 1 | 0 | 0 | 0 | 1 |
| 11 | 0 | 0 | 0 | 0 | 0 | 0 | 0 | 0 | 0 | 1 |
| 12 | 0 | 0 | 0 | 0 | 0 | 1 | 0 | 0 | 0 | 1 |
| 13 | 0 | 0 | 0 | 0 | 1 | 1 | 0 | 0 | 0 | 0 |
| 14 | 0 | 0 | 0 | 0 | 0 | 1 | 0 | 0 | 0 | 1 |
| 15 | 0 | 0 | 0 | 0 | 0 | 1 | 0 | 0 | 0 | 0 |
| 16 | 0 | 0 | 0 | 0 | 0 | 1 | 0 | 0 | 0 | 1 |
| 17 | 0 | 0 | 0 | 0 | 0 | 1 | 0 | 0 | 0 | 1 |
| 18 | 0 | 0 | 0 | 0 | 0 | 0 | 0 | 0 | 2 | 0 |
| 19 | 0 | 0 | 0 | 0 | 0 | 1 | 0 | 0 | 0 | 1 |
| 20 | 0 | 0 | 0 | 0 | 0 | 0 | 0 | 0 | 0 | 1 |
| 21 | 0 | 0 | 0 | 0 | 0 | 0 | 0 | 0 | 0 | 0 |
| 22 | 0 | 0 | 0 | 0 | 0 | 0 | 0 | 0 | 0 | 0 |
| 23 | 0 | 0 | 0 | 0 | 0 | 0 | 0 | 0 | 0 | 0 |
| 24 | 0 | 0 | 0 | 0 | 0 | 0 | 0 | 0 | 4 | 0 |
| 25 | 0 | 0 | 0 | 0 | 0 | 0 | 0 | 0 | 0 | 0 |
| 26 | 0 | 0 | 0 | 0 | 0 | 0 | 0 | 0 | 0 | 0 |
| 27 | 0 | 0 | 0 | 0 | 0 | 2 | 0 | 0 | 0 | 0 |
| 28 | 0 | 0 | 0 | 0 | 0 | 1 | 0 | 0 | 0 | 0 |
| 29 | 0 | 0 | 0 | 0 | 0 | 0 | 0 | 0 | 0 | 0 |
| 30 | 0 | 0 | 0 | 0 | 0 | 2 | 0 | 0 | 0 | 0 |
| 31 | 0 | 0 | 0 | 0 | 0 | 1 | 0 | 0 | 0 | 0 |
| 32 | 0 | 0 | 0 | 0 | 0 | 2 | 0 | 0 | 0 | 0 |
| 33 | 0 | 0 | 0 | 0 | 0 | 1 | 0 | 0 | 0 | 0 |
| 34 | 0 | 0 | 0 | 0 | 0 | 0 | 0 | 0 | 0 | 0 |
| 35 | 0 | 0 | 0 | 0 | 0 | 0 | 0 | 0 | 0 | 0 |

| **D. Trial by trial USVs emission during ITI**  Individual data points showing the USV emitted during each trial of extinction training (%) | | | | | | | | | |  |
| --- | --- | --- | --- | --- | --- | --- | --- | --- | --- | --- |
| **Trial** | **Control Group** | | | | | | | | |  |
| 1 | 0 | 0 | 0 | 0 | 0 | 0 | 0 | 0 | 0 |  |
| 2 | 0 | 0 | 0 | 0 | 0 | 0 | 0 | 0 | 0 |  |
| 3 | 0 | 0 | 0 | 0 | 0 | 0 | 0 | 0 | 0 |  |
| 4 | 0 | 0 | 0 | 1 | 0 | 0 | 0 | 0 | 0 |  |
| 5 | 0 | 0 | 0 | 11 | 0 | 0 | 0 | 0 | 0 |  |
| 6 | 0 | 0 | 0 | 17 | 0 | 0 | 1 | 0 | 0 |  |
| 7 | 0 | 0 | 0 | 14 | 0 | 0 | 7 | 0 | 0 |  |
| 8 | 0 | 1 | 0 | 6 | 0 | 0 | 5 | 0 | 0 |  |
| 9 | 0 | 18 | 0 | 10 | 0 | 0 | 5 | 0 | 0 |  |
| 10 | 0 | 21 | 0 | 10 | 0 | 0 | 3 | 0 | 0 |  |
| 11 | 0 | 19 | 0 | 10 | 0 | 0 | 3 | 0 | 0 |  |
| 12 | 0 | 15 | 3 | 8 | 0 | 0 | 0 | 0 | 0 |  |
| 13 | 0 | 11 | 8 | 8 | 0 | 0 | 0 | 0 | 0 |  |
| 14 | 0 | 12 | 8 | 10 | 0 | 0 | 3 | 0 | 0 |  |
| 15 | 0 | 12 | 0 | 6 | 0 | 0 | 3 | 0 | 0 |  |
| 16 | 0 | 16 | 0 | 5 | 0 | 0 | 0 | 0 | 0 |  |
| 17 | 0 | 18 | 0 | 0 | 0 | 0 | 0 | 0 | 0 |  |
| 18 | 0 | 15 | 0 | 0 | 0 | 0 | 0 | 0 | 0 |  |
| 19 | 0 | 17 | 0 | 0 | 0 | 0 | 0 | 0 | 0 |  |
| 20 | 0 | 17 | 0 | 0 | 0 | 0 | 0 | 0 | 0 |  |
| 21 | 0 | 11 | 0 | 0 | 0 | 0 | 0 | 0 | 0 |  |
| 22 | 0 | 9 | 0 | 0 | 0 | 0 | 0 | 0 | 0 |  |
| 23 | 0 | 9 | 0 | 0 | 0 | 0 | 0 | 0 | 0 |  |
| 24 | 0 | 6 | 0 | 0 | 0 | 0 | 0 | 0 | 0 |  |
| 25 | 0 | 9 | 0 | 0 | 0 | 0 | 0 | 0 | 0 |  |
| 26 | 0 | 7 | 0 | 0 | 0 | 0 | 0 | 0 | 0 |  |
| 27 | 0 | 12 | 0 | 0 | 0 | 0 | 0 | 0 | 0 |  |
| 28 | 0 | 11 | 0 | 0 | 0 | 0 | 0 | 0 | 0 |  |
| 29 | 0 | 7 | 0 | 0 | 0 | 0 | 0 | 0 | 0 |  |
| 30 | 0 | 10 | 0 | 0 | 0 | 0 | 0 | 0 | 0 |  |
| 31 | 0 | 7 | 0 | 0 | 0 | 0 | 0 | 0 | 0 |  |
| 32 | 0 | 9 | 0 | 0 | 0 | 0 | 0 | 0 | 0 |  |
| 33 | 0 | 3 | 0 | 0 | 0 | 0 | 0 | 0 | 0 |  |
| 34 | 0 | 7 | 0 | 0 | 0 | 0 | 0 | 0 | 0 |  |
| 35 | 0 | 7 | 0 | 0 | 0 | 0 | 0 | 0 | 0 |  |
| **Trial** | **DREADD Group** | | | | | | | | |  |
| 1 | 0 |  | 0 | 0 | 0 | 1 | 0 | 0 | 0 | 2 |
| 2 | 0 |  | 0 | 0 | 0 | 4 | 0 | 0 | 0 | 9 |
| 3 | 0 |  | 0 | 0 | 0 | 9 | 0 | 0 | 0 | 8 |
| 4 | 0 |  | 0 | 0 | 0 | 2 | 0 | 3 | 0 | 7 |
| 5 | 0 |  | 0 | 0 | 0 | 0 | 0 | 0 | 0 | 10 |
| 6 | 0 |  | 0 | 0 | 0 | 0 | 0 | 0 | 0 | 8 |
| 7 | 0 |  | 0 | 0 | 0 | 0 | 0 | 3 | 0 | 9 |
| 8 | 0 |  | 0 | 0 | 0 | 2 | 0 | 0 | 0 | 6 |
| 9 | 0 |  | 0 | 0 | 0 | 4 | 0 | 0 | 0 | 8 |
| 10 | 0 |  | 0 | 0 | 0 | 2 | 0 | 0 | 0 | 9 |
| 11 | 0 |  | 0 | 0 | 0 | 4 | 0 | 0 | 0 | 6 |
| 12 | 13 |  | 0 | 0 | 2 | 5 | 0 | 0 | 0 | 9 |
| 13 | 6 |  | 0 | 0 | 4 | 6 | 0 | 0 | 0 | 6 |
| 14 | 0 |  | 0 | 0 | 7 | 3 | 0 | 0 | 0 | 6 |
| 15 | 0 |  | 0 | 0 | 6 | 3 | 0 | 0 | 0 | 5 |
| 16 | 0 |  | 0 | 0 | 5 | 3 | 0 | 0 | 0 | 7 |
| 17 | 0 |  | 0 | 0 | 6 | 2 | 0 | 0 | 1 | 7 |
| 18 | 0 |  | 0 | 0 | 3 | 3 | 0 | 0 | 0 | 5 |
| 19 | 0 |  | 0 | 0 | 3 | 4 | 0 | 0 | 0 | 5 |
| 20 | 0 |  | 0 | 0 | 6 | 3 | 0 | 0 | 0 | 4 |
| 21 | 0 |  | 0 | 0 | 4 | 0 | 0 | 0 | 0 | 3 |
| 22 | 0 |  | 0 | 0 | 0 | 2 | 0 | 0 | 0 | 0 |
| 23 | 0 |  | 0 | 0 | 0 | 0 | 0 | 0 | 0 | 0 |
| 24 | 0 |  | 0 | 0 | 0 | 0 | 0 | 0 | 4 | 0 |
| 25 | 0 |  | 0 | 0 | 0 | 0 | 0 | 0 | 0 | 0 |
| 26 | 0 |  | 0 | 0 | 0 | 0 | 0 | 0 | 0 | 0 |
| 27 | 0 |  | 0 | 0 | 0 | 3 | 0 | 0 | 0 | 0 |
| 28 | 0 |  | 0 | 0 | 0 | 2 | 0 | 0 | 0 | 0 |
| 29 | 0 |  | 0 | 0 | 0 | 3 | 0 | 0 | 0 | 0 |
| 30 | 0 |  | 0 | 0 | 0 | 0 | 0 | 0 | 0 | 0 |
| 31 | 0 |  | 0 | 0 | 0 | 6 | 0 | 0 | 0 | 0 |
| 32 | 0 |  | 0 | 0 | 0 | 2 | 0 | 0 | 0 | 0 |
| 33 | 0 |  | 0 | 0 | 0 | 0 | 0 | 0 | 0 | 0 |
| 34 | 0 |  | 0 | 0 | 0 | 0 | 0 | 0 | 0 | 0 |
| 35 | 0 |  | 0 | 0 | 0 | 0 | 0 | 0 | 0 | 0 |
